# Risk of recurrence after local resection of T1 rectal cancer: a meta-analysis with meta-regression

**DOI:** 10.1007/s00464-022-09396-3

**Published:** 2022-06-30

**Authors:** Nik Dekkers, Hao Dang, Jolein van der Kraan, Saskia le Cessie, Philip P. Oldenburg, Jan W. Schoones, Alexandra M. J. Langers, Monique E. van Leerdam, Jeanin E. van Hooft, Yara Backes, Katarina Levic, Alexander Meining, Giorgio M. Saracco, Fabian A. Holman, Koen C. M. J. Peeters, Leon M. G. Moons, Pascal G. Doornebosch, James C. H. Hardwick, Jurjen J. Boonstra

**Affiliations:** 1 Department of Gastroenterology and Hepatology, Leiden University Medical Center, Albinusdreef 2, 2333 ZA Leiden, The Netherlands; 2 Department of Biomedical Data Sciences, Leiden University Medical Center, Leiden, The Netherlands; 3 Directorate of Research Policy (Formerly: Walaeus Library), Leiden University Medical Center, Leiden, The Netherlands; 4 Department of Gastroenterology and Hepatology, University Medical Center Utrecht, Utrecht, The Netherlands; 5 Gastrounit-Surgical Division, Center for Surgical Research, Copenhagen University Hospital Hvidovre, Copenhagen, Denmark; 6 Department of Gastroenterology, University Hospital of Würzburg, Würzburg, Germany; 7 Division of Gastroenterology, Department of Medical Sciences, Molinette Hospital, University of Turin, Turin, Italy; 8 Department of Surgery, Leiden University Medical Center, Leiden, The Netherlands; 9 Department of Surgery, IJsselland Hospital, Capelle Aan Den IJssel, The Netherlands

**Keywords:** T1 rectal cancer, Local surgical resection, Therapeutic endoscopy, Follow-up, Recurrence

## Abstract

**Background:**

T1 rectal cancer (RC) patients are increasingly being treated by local resection alone but uniform surveillance strategies thereafter are lacking. To determine whether different local resection techniques influence the risk of recurrence and cancer-related mortality, a meta-analysis was performed.

**Methods:**

A systematic search was conducted for T1RC patients treated with local surgical resection. The primary outcome was the risk of RC recurrence and RC-related mortality. Pooled estimates were calculated using mixed-effect logistic regression. We also systematically searched and evaluated endoscopically treated T1RC patients in a similar manner.

**Results:**

In 2585 unique T1RC patients (86 studies) undergoing local surgical resection, the overall pooled cumulative incidence of recurrence was 9.1% (302 events, 95% CI 7.3–11.4%; *I*^2^ = 68.3%). In meta-regression, the recurrence risk was associated with histological risk status (*p* < 0.005; low-risk 6.6%, 95% CI 4.4–9.7% vs. high-risk 28.2%, 95% CI 19–39.7%) and local surgical resection technique (*p* < 0.005; TEM/TAMIS 7.7%, 95% CI 5.3–11.0% vs. other local surgical excisions 10.8%, 95% CI 6.7–16.8%). In 641 unique T1RC patients treated with flexible endoscopic excision (16 studies), the risk of recurrence (7.7%, 95% CI 5.2–11.2%), cancer-related mortality (2.3%, 95% CI 1.1–4.9), and cancer-related mortality among patients with recurrence (30.0%, 95% CI 14.7–49.4%) were comparable to outcomes after TEM/TAMIS (risk of recurrence 7.7%, 95% CI 5.3–11.0%, cancer-related mortality 2.8%, 95% CI 1.2–6.2% and among patients with recurrence 35.6%, 95% CI 21.9–51.2%).

**Conclusions:**

Patients with T1 rectal cancer may have a significantly lower recurrence risk after TEM/TAMIS compared to other local surgical resection techniques. After TEM/TAMIS and endoscopic resection the recurrence risk, cancer-related mortality and cancer-related mortality among patients with recurrence were comparable. Recurrence was mainly dependent on histological risk status.

**Graphical abstract:**

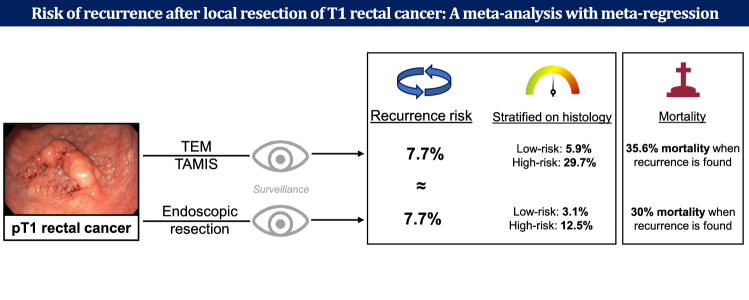

**Supplementary Information:**

The online version contains supplementary material available at 10.1007/s00464-022-09396-3.

The introduction of population-based screening has resulted in an increased number of early invasive, or T1, rectal cancers (T1RC) [1]. Over the last years, a shift can be observed from major surgery towards local, organ-preserving endoscopic or surgical resection techniques as primary treatment for these tumors.

The decision whether to perform additional total mesorectal excision (TME) after local resection mainly depends on the oncological risk (which is based on histological high-risk features for lymph node metastasis (LNM) [2]), operative risk and patient preferences. Considering the limited accuracy of the histological risk stratification models, and the significant morbidity and decrease in quality of life that are associated with TME, there has been an increased tendency towards close-surveillance strategies after local resection of T1RC [3–6].

Surveillance after local resection of T1RC is currently quite heterogeneous [7], and needs to be optimized to improve the efficacy of surveillance. To determine the optimal surveillance strategy, it is important to determine the risk, type and prognosis of cancer recurrences that could occur. This meta-analysis aims to estimate the cumulative incidence of RC recurrence and RC-related mortality for patients with local surgically resected T1RC and to compare this with results of endoscopically treated T1RC patients.

## Materials and methods

This meta-analysis was reported according to the Preferred Reporting Items for Systematic Reviews and Meta-Analysis statement [8]. Information regarding the search strategy, data extraction, definitions and classifications, and risk of bias assessment can be found in the Supplementary methods. Approval of the institutional review board (IRB) and written consent was not needed.

### Selection criteria for local surgical resection

A systematic literature search was conducted in PubMed, Embase, Web of Science and Cochrane Library from inception until May 19, 2021. Inclusion criteria were: 1. histologically confirmed pT1RCs 2. local surgical resection alone, 3. the proportion of recurrences after local surgical resection of T1RCs was reported 4. original peer-reviewed articles. Exclusion criteria were: 1. prior or additional therapy (e.g., endoscopic resection, oncological surgery, chemotherapy or radiotherapy), 2. hereditary predisposition for CRC, 3. inflammatory bowel disease, 4. studies with < 5 patients with T1RC undergoing local surgical resection, 5. studies without original patient data (e.g., reviews or meta-analyses), 6. conference abstracts, 7. animal studies and 8. non-English or non-German articles. In case of overlapping cohorts, the cohort with the largest number of patients, or covering the largest period of time was selected.

T1RCs were defined as rectal tumors with histologic tumor invasion through the muscularis mucosae and into, but not beyond, the submucosa. Local surgical resection was defined as any type of local resection that was used to excise a rectal tumor without lymph node dissection, and that did not use flexible endoscopy (i.e., no endoscopic submucosal dissection (ESD), endoscopic full-thickness resection (eFTR), endoscopic mucosal resection (EMR), or snare polypectomy). High-risk criteria for LNM defined by The Japanese Society for Cancer of the Colon and Rectum (JSCCR) include: positive resection margins, deep submucosal invasion, grade 3 differentiation, lymphovascular invasion and high-grade tumor budding [2].

### Selection criteria for local endoscopic resection

Data of endoscopically treated T1RC patients were extracted from our previous meta-analysis on recurrences after local endoscopic resection of T1 colorectal cancer[9]. This search was updated until May 19, 2021 and additional data regarding primary outcomes or main study characteristics for the subgroup of T1RC patients were requested from the corresponding authors. The in- and exclusion criteria of the current analysis were similar to those of the previous analysis [9], except for treatment and location (Supplementary Table 1).

### Data acquisition

Data extraction and risk of bias assessment were independently performed by 3 authors (ND, HD, PO). In case of disagreement without consensus after discussion, a fourth assessor (JB) was decisive. Relevant study-level parameters and individual patient-level data of recurrence cases were extracted. The risk of bias was assessed using a modified Newcastle–Ottawa Scale [10]. An additional random data check was performed by the decisive assessor to ensure the data quality.

### Study outcomes

The primary outcome was the cumulative incidence of RC recurrence (locoregional or distant) and RC-related mortality during follow-up. Locoregional recurrence was defined as endoluminal cancer at the primary resection site or pelvic LNM. Distant recurrence was defined as any metastasis outside the pelvic area. Secondary outcomes were the cumulative incidence of locoregional RC recurrence only, any locoregional RC recurrence and any distant recurrence.

### Statistical analysis

All analyses were performed in R v4.1.0 [11] using the package *metafor v3.0.1* [12]. Cumulative incidences of all study outcomes were modeled on the logit scale using mixed-effects logistic regression (13). Thereafter, results were transformed back to proportions and presented as point estimates with 95%-confidence intervals (95% CI). The risk of publication bias was examined using a funnel plot with the square root of the study size on the y-axis [14].

Statistical heterogeneity was quantified using *I*^2^ statistic and tau-squared (*τ*^2^). Univariable meta-regression and subgroup analyses were performed to explore possible sources of heterogeneity with predefined potential predictors: study characteristics (e.g., publication year, study design), individual items from the risk of bias assessment, follow-up characteristics (e.g., duration and intensity), and clinical characteristics (e.g., resection technique, histology). Only studies with subgroups of ≥ 5 patients, for whom the exact number of events could be determined, were included in meta-regression and subgroup analyses. Meta-regression was only performed when at least 10 studies could be analysed [15]. *p* values < 0.05 were considered statistically significant.

## Results

### Study characteristics for local surgical resection

**Fig. 1:**
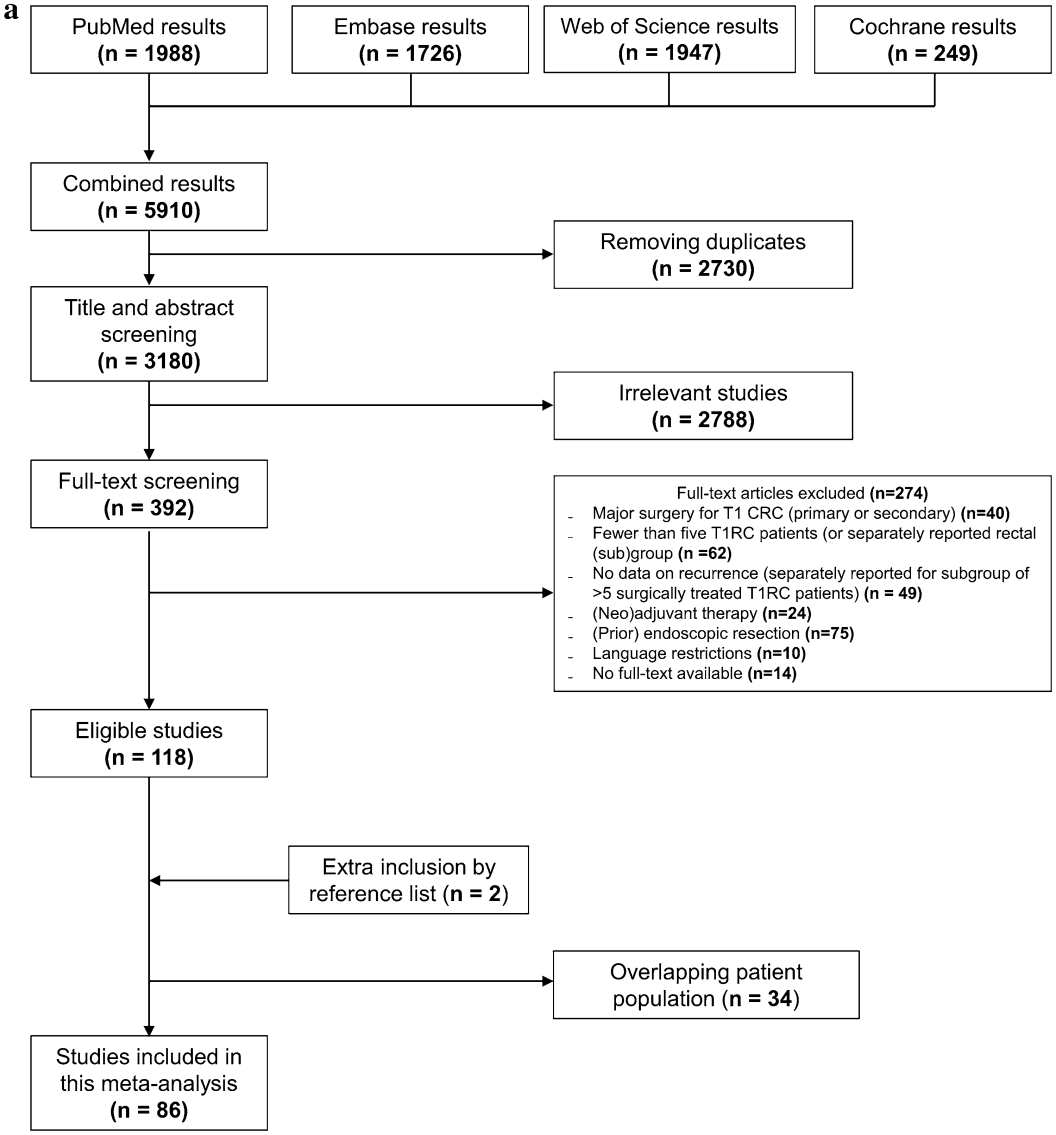

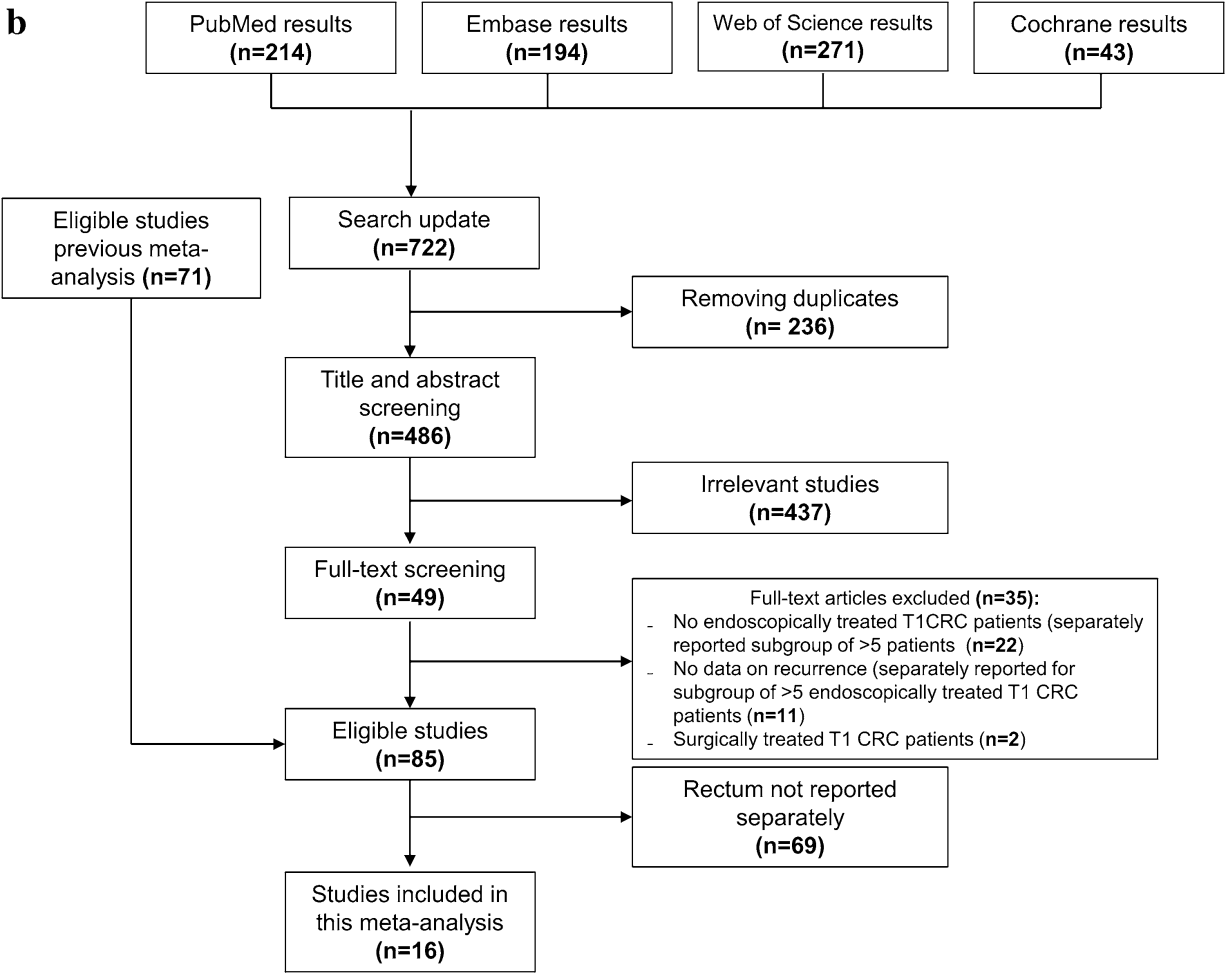
Flow diagram of the selection process for studies on local surgical resection (**a**) and endoscopic resection (**b**). *(C)RC* (colo)rectal cancer

Our search identified 5910 articles, of which 86 reported unique patient cohorts and were included (Fig. 1a) [16–101]. These studies consisted of 2585 patients undergoing local surgical resection for T1RC, with data on the cumulative incidence of recurrence. Eighty-five studies also reported separate incidences of locoregional and distant recurrences.

The extracted data and risk of bias assessment of the included studies are shown in Supplementary analyses. Most studies were performed in Europe (46 studies, *n* = 1506 patients), followed by North America (20 studies, *n* = 608), Asia (17 studies, *n* = 438), South America (2 studies, *n* = 15) and Australia (one study, *n* = 618). No obvious asymmetry was observed in the funnel plot (Supplementary Fig. 1).

**Fig. 2:**
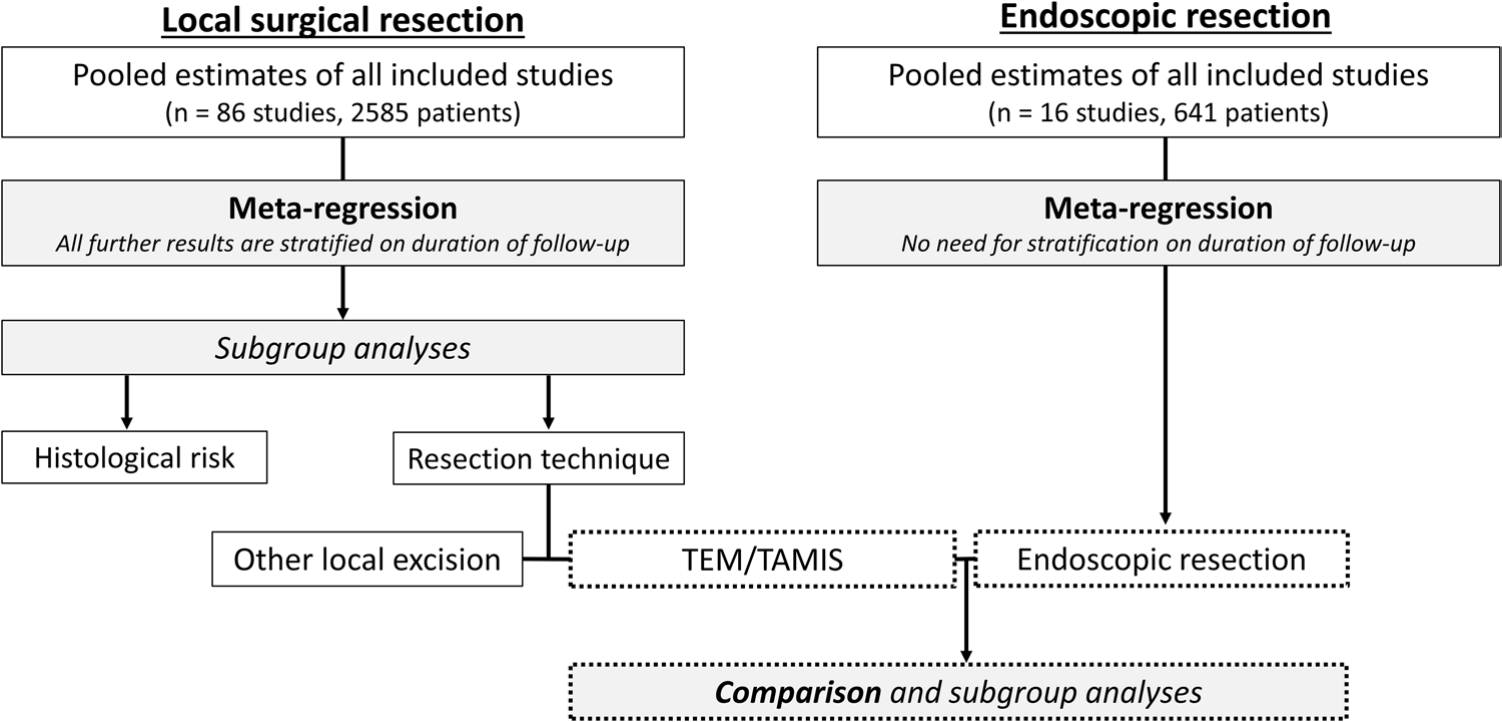
Flow diagram of the study process. *TEM* transanal endoscopic microsurgery, *TAMIS* transanal minimally invasive surgery

In 41 studies the transanal endoscopic microsurgery (TEM) technique was investigated and transanal minimally invasive surgery (TAMIS) in 4. The majority of patients in the remaining 41 studies underwent other local surgical resection techniques with direct visualization; these were grouped as “local excision” (LE; e.g., Park method or using the Ferguson anoscope). Fifty-five studies reported data on the resection plane; almost all patients in these studies underwent a full-thickness resection (99.2%). The mean and minimum follow-up could be determined in 15 (range, 18.2–72.5) and 51 studies (range 1–60), respectively. Complete data on follow-up schemes (i.e., which follow-up modalities and intervals per modality) was reported in 42 studies; schemes were classified as “not strict” in 6 (14.3%), “strict” in 13 (31.0%), and “very strict” in 23 studies (54.8%). The definitions used for these groups are shown in the Supplementary methods. A flow diagram of the study process is shown in Fig. 2.

### Pooled estimates of all included studies

**Fig. 3:**
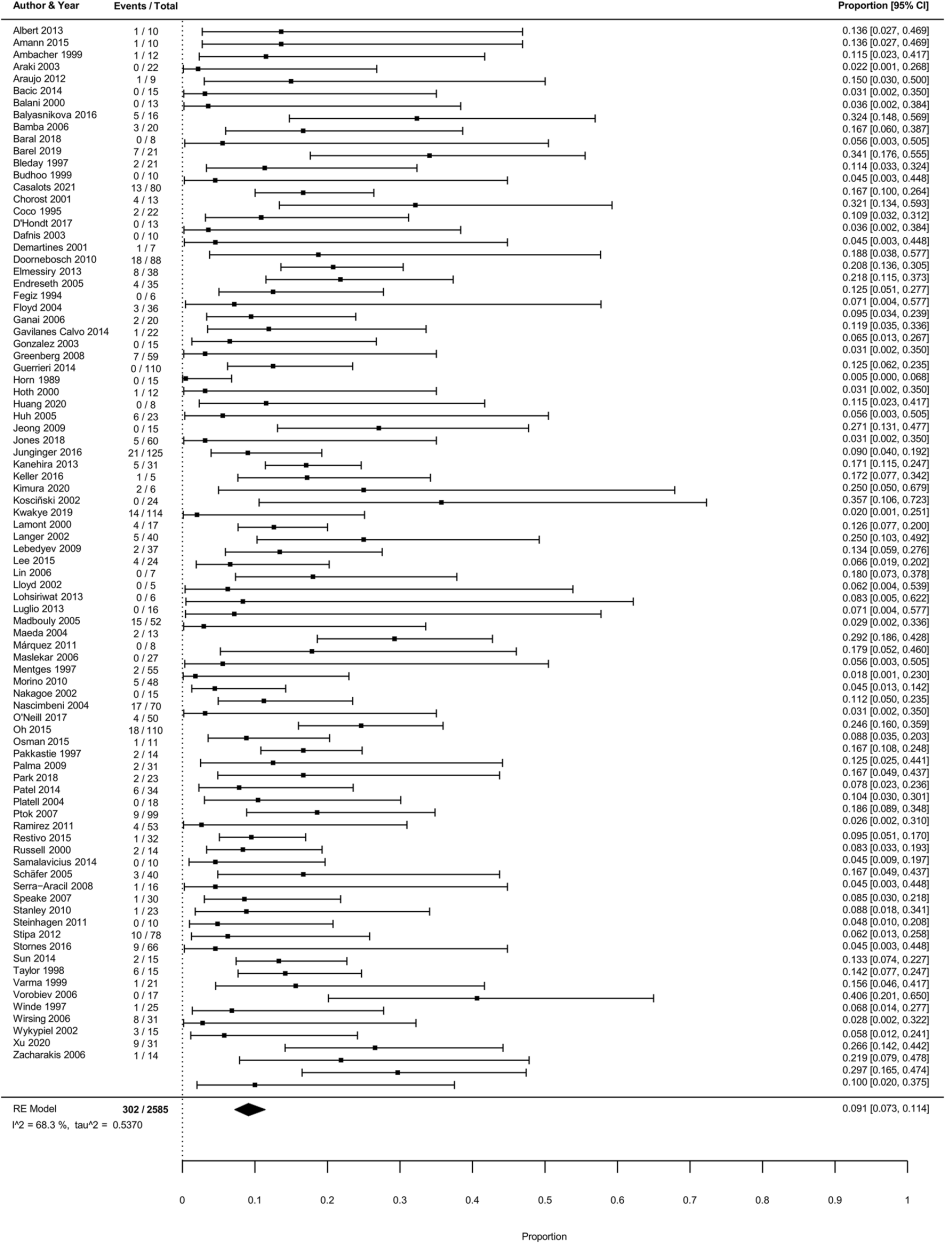
Forest plot with cumulative incidences of any RC recurrence after local surgical resection. To visualize incidence estimates of studies with 0 events, a continuity correction of + 0.5 was applied. Values of the pooled estimates, *I*^2^ and *τ*^2^ are calculated using a model without continuity correction

Overall, 302 out of 2585 patients experienced recurrence after local surgical resection. The pooled cumulative incidence of any RC recurrence was 9.1% (95% CI 7.3–11.4%; *I*^2^ = 68.3%; Fig. 3).

### Meta-regression

In meta-regression, histological risk status (low-risk vs. high-risk) and, local surgical resection technique (TEM/TAMIS vs. other local surgical excisions) were associated with the risk of recurrence (Supplementary Tables 2 and 3). Therefore, subgroup analyses were performed for histological risk status and local surgical resection technique. Further analyses were stratified according to the duration of follow-up because risk of recurrence increased with longer mean follow-up duration (Supplementary Table 2). Results for studies with ≥ 2-year follow-up are shown below; results for all studies, for studies with a ≥ 5 years follow-up and detailed information regarding the meta-regression results are shown in Supplementary results.

### Pooled estimates of studies with ≥ 2 years follow-up

The pooled cumulative incidence of any RC recurrence was 9.2% (194/1713 events; 95% CI 7.1–11.9%; *I*^2^ = 60.8%; Supplementary Fig. 2). Pooled incidences of all secondary outcomes are shown in Supplementary analyses. The pooled incidence of RC-related mortality was 1.9% (31/898 events, 27 studies; 95% CI 0.9–4.2%; *I*^2^ = 69.3%; Supplementary Fig. 3). The RC-related mortality rate among patients with recurrence was 28.7% (31/108). All of these patients died of disease progression.

### Subgroup analyses in studies with ≥ 2 years follow-up

#### Low-risk versus high-risk

**Fig. 4:**
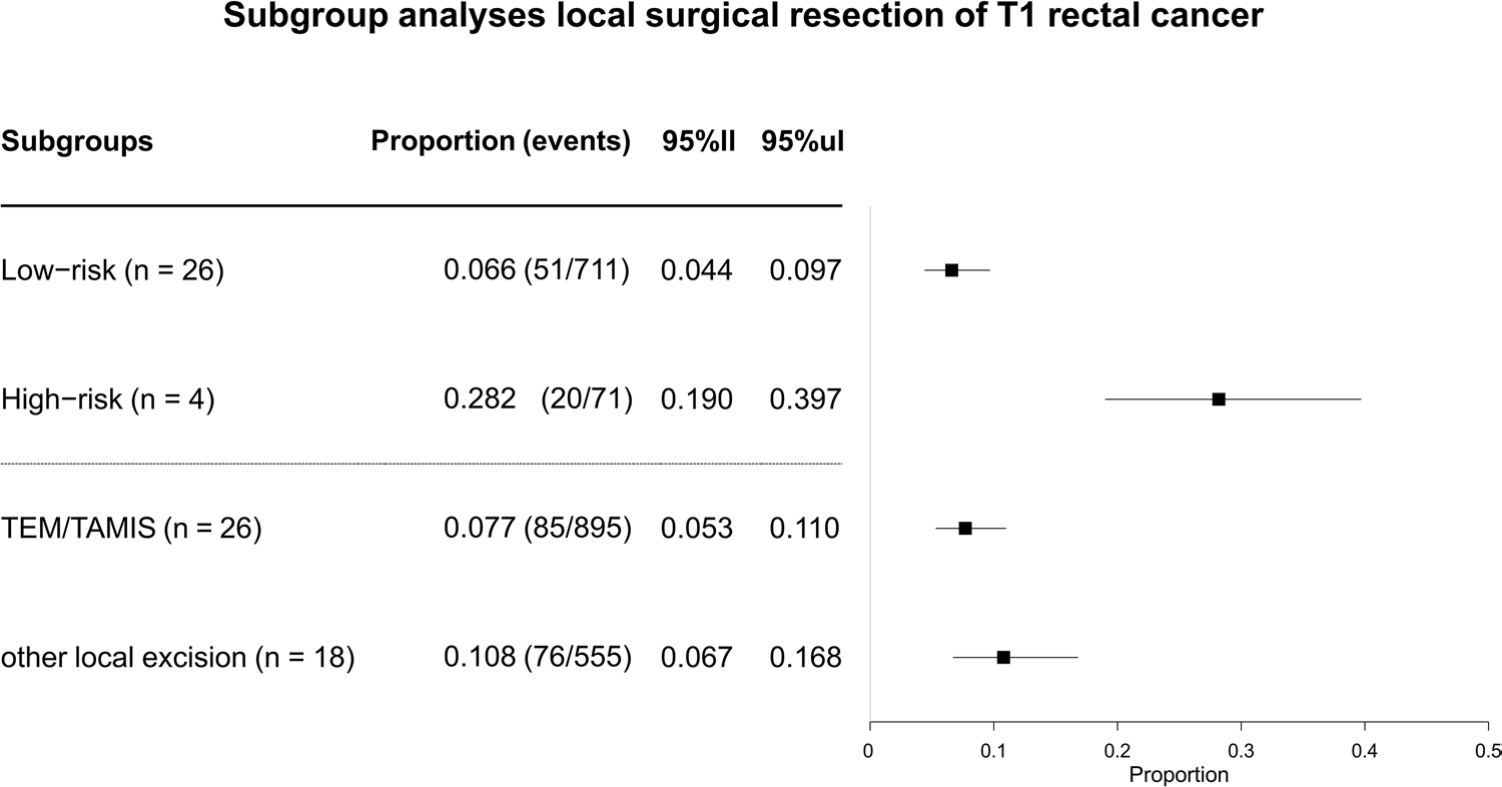
Forest plot with cumulative incidences of any RC recurrence after local surgical resection with subgroups based on histological risk status and local surgical resection technique. *95%ll* 95% confidence interval lower limit, *95%ul* 95% confidence interval upper limit, *TEM* transanal endoscopic microsurgery, *TAMIS* transanal minimally invasive surgery

Twenty-six studies reported a subgroup of ≥ 5 patients with low-risk T1RC and sufficient data on recurrence, and 4 studies did so for high-risk T1RCs. The definitions of low- and high-risk T1RCs were diverse. Most studies used 3 risk criteria: differentiation grade was used the most and tumor budding the least (Supplementary Fig. 4). The cumulative incidence of any RC recurrence was 6.6% for low-risk T1RCs (51/711 events; 95% CI 4.4–9.7%; *I*^2^ = 22.4%) and 28.2% for high-risk T1RCs (20/71 events; 95% CI 19–39.7%; *I*^2^ = 0.0%) (Fig. 4).

#### TEM/TAMIS versus local excision

The cumulative incidence of RC recurrence was 7.7% after TEM/TAMIS (85/895 events; 95% CI 5.3–11.0%; *I*^2^ = 47.7%) and 10.8% after local surgical excision techniques with direct visualization (76/555 events; 95% CI 6.7–16.8%; *I*^2^ = 65.3%) (Fig. 4). This difference was mainly due to an increased incidence of endoluminal local-site recurrences; 4.7% for the TEM/TAMIS (50/859 events; 95% CI 2.9–7.6%; *I*^2^ = 44.2%) and 7.2% for local excision (38/480 events; 95% CI 4.2–12%; *I*^2^ = 29.9%). This subgroup analyses confirmed that TEM/TAMIS is superior to other local surgical excision techniques with regard to recurrence. Outcomes of the TEM/TAMIS technique will therefore be compared to the endoscopic data. Secondary outcomes for all subgroup analyses are detailed in Supplementary results and Supplementary analyses.

### TEM/TAMIS versus endoscopic resection

**Fig. 5:**
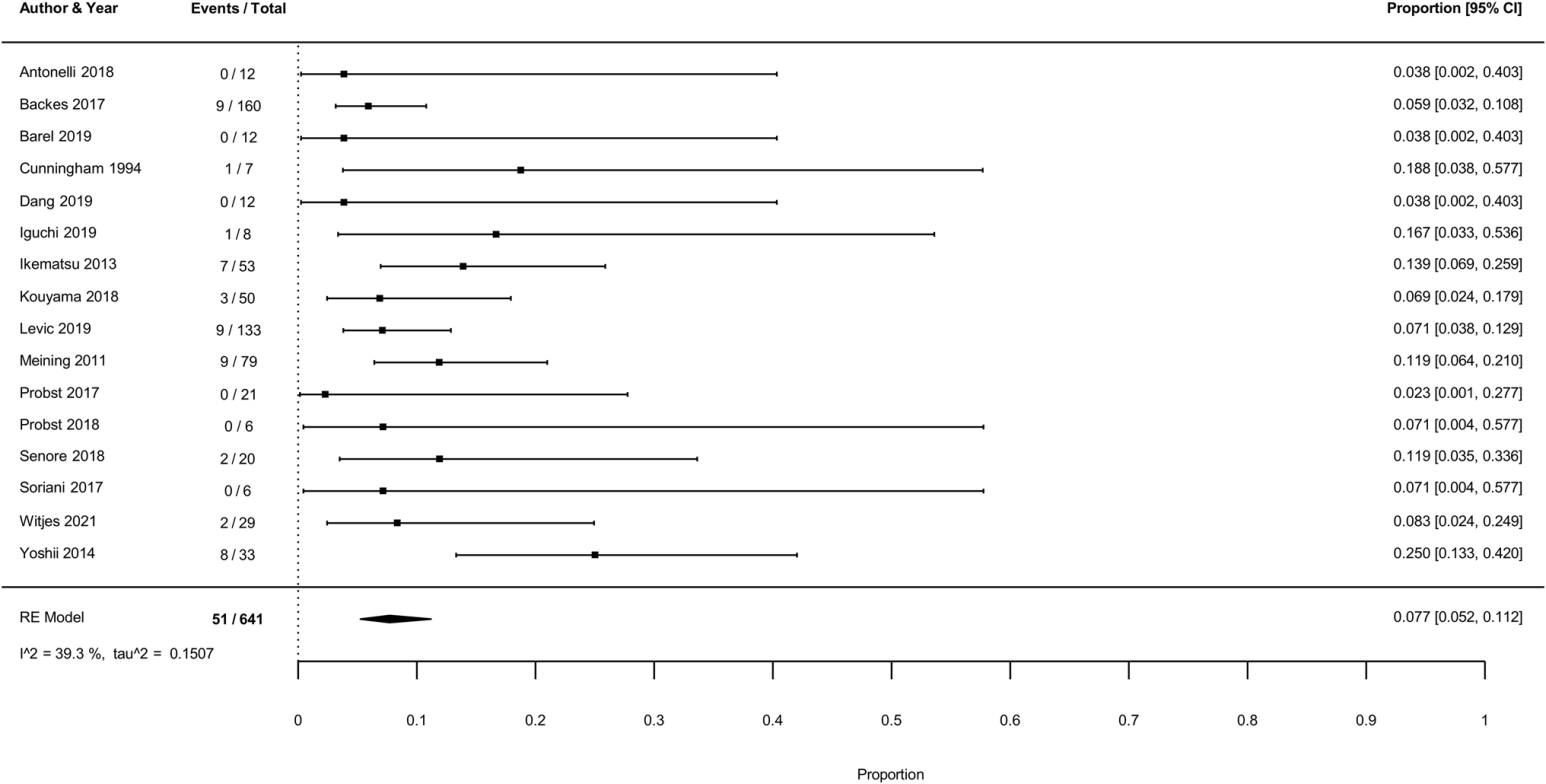
Forest plot with cumulative incidences of any RC recurrence after endoscopic resection. To visualize incidence estimates of studies with 0 events, a continuity correction of + 0.5 was applied. Values of the pooled estimates, *I*^2^ and *τ*^2^ are calculated using a model without continuity correction

The previous meta-analysis and search update yielded 16 eligible studies with 641 patients and 51 recurrences (Fig. 1B, Supplementary analyses) [26, 102–116]. The studied endoscopic resection techniques included ESD, eFTR, EMR, and snaring polypectomy. “Very strict” follow-up schemes were reported in 50.0% (13/26) of the TEM/TAMIS studies and in 37% (10/27) of the endoscopic studies (Supplementary Fig. 5). The pooled incidence of RC recurrence was comparable between endoscopically treated (7.7%; 95% CI 5.2–11.2%; *I*^2^ = 39.3%; Fig. 5) and TEM/TAMIS-treated patients with ≥ 2 years follow-up (7.7%). Also after correcting for the proportion of low- and high-risk T1RCs, meta-regression showed no statistical difference between TEM/TAMIS and endoscopic resection (p = 0.244).RC-related mortality was also comparable between endoscopically treated (2.3%; 95% CI 1.1–4.9%; *I*^2^ = 18.4%) and TEM/TAMIS-treated patients (2.8%; 95% CI 1.2–6.2%; *I*^2^ = 48.9%) and among the recurrence cases (30.0% versus 35.6%, respectively). The timing of the recurrences after endoscopic resection is shown in Supplementary Fig. 6. The overall pooled incidence of RC recurrence after TEM/TAMIS and endoscopic resections combined was 7.7% (136/1536 events, 95% CI 5.9–10.0%; *I*^2^ = 46.2%).

The risk of recurrence for low-risk T1RC was 5.9% after TEM/TAMIS (27/406 events, 95% CI 3.4–10.0%; *I*^2^ = 24.1%) and 3.1% after endoscopic resection (4/128 events, 95% CI 1.2–8.0%; *I*^2^ = 0.0%). Twenty-four of these low-risk recurrences were endoluminal (2 with synchronous locoregional LNM; 9 also presented with distant metastasis at the time of the local recurrence or later), the other 7 were distant metastasis. For 29 of the 31 low-risk T1RC recurrences it was stated that the local resection was complete. For the other 2 recurrence cases this was not stated explicitly. For high-risk T1RCs the risk of recurrence was 29.7% after TEM/TAMIS (11/37 events, 95% CI 17.3–46.1%; *I*^2^ = 0.0%) and 12.5% after endoscopic resection (25/200 events, 95% CI 8.6–17.8%; *I*^2^ = 0.0%). In 29 of the 43 studies on local endoscopic resections for T1RC, 4–5 JSCCR risk criteria were used; for studies on TEM/TAMIS this was 5 of the 33 studies (Supplementary Fig. 4). Other secondary outcomes are shown in Supplementary results*.*

## Discussion

This meta-analysis is the first to meticulously analyze the long-term outcomes of T1RC patients treated by local surgical resection, and to relate these outcomes to those of endoscopically treated T1RC patients. The overall recurrence risk after local surgical resection of T1RC was found to be around 9%.

Meta-regression analysis demonstrated that the risk of recurrence was significantly affected by several factors, including resection technique. In line with previous studies [117], our subgroup analyses confirmed that TEM/TAMIS (7.7%) is superior to other local surgical excision techniques using direct visualization (10.8%) with regard to recurrence. Although TEM/TAMIS were introduced later, it is unlikely that this biased the results because meta-regression showed no association between publication year and risk of recurrence. Instead, the difference could mainly be attributed to an increased risk of endoluminal local-site recurrences. This suggests that the oncological superiority of TEM/TAMIS is most likely explained by the camera-assisted visualization, and the use of a pneumorectum, which allows for improved visualization of tumor margins and increases the chance of achieving a complete resection. Tumor height may also have influenced the outcome of local surgical resections. Unfortunately data on tumor height was scarcely reported and could not always be extracted for the correct subgroup, therefore it was not possible to further stratify our results.

Another factor that significantly influenced the recurrence risk was histological risk status. This was in accordance with findings of our previous meta-analysis [9]. In subgroup analyses the difference between low- and high-risk tumors was confirmed for both TEM/TAMIS-treated (5.9% recurrence risk for low-risk T1RC vs. 29.7% for high-risk T1RC) and endoscopically treated patients (3.1% recurrence risk for low-risk T1RC vs. 12.5% for high-risk T1RC). There appears to be a difference in the risk of recurrence for high-risk T1RC treated by TEM/TAMIS or endoscopic resection (TEM/TAMIS: 11/37 events in 2 studies, endoscopic resection: 25/200 events in 8 studies). Due to the limited number of studies included in this subgroup analysis, it was not possible to draw any valid conclusions on these findings.

When comparing TEM/TAMIS to endoscopic resections, we observed that overall recurrence rates (7.7% and 7.7%, respectively), RC-related mortality rates (2.8% and 2.3%, respectively) and mortality rates among recurrences (35.6% and 30.0%, respectively) were quite similar. A randomized non-inferiority trial is pending to confirm these results [118]. Despite the similarities in oncological outcomes, we found that risk stratification and follow-up varied considerably between local surgically and endoscopically treated T1RC patients. Firstly, the number of JSCCR criteria used for risk stratification were quite different, which makes it difficult to compare recurrence risks stratified by histology. Two-third of studies on endoscopic resections used > 3 criteria to define high-risk tumors, but among studies on TEM/TAMIS only ~ 15% used > 3 criteria. This has most probably caused an overestimation of the recurrence risk in the group of TEM/TAMIS-treated low-risk T1RC, as some of these patients would have been classified as high-risk if more JSCCR criteria had been used. However, it was impossible to draw any valid conclusions on the clinical relevance of each high-risk criterion from these results, as the available data did not allow us to study the criteria individually. More universal histological assessment of T1RC by a dedicated pathologist is therefore warranted. Secondly, the reported follow-up schemes of TEM/TAMIS-treated T1RC patients were often much stricter than the schemes of endoscopically treated patients (Supplementary Fig. 6), but compliance to these schemes were rarely reported. Considering the comparable outcomes of TEM/TAMIS and endoscopic resection, it appears that at a certain point further intensifying the follow-up, using current follow-up modalities, might not necessarily lead to increased detection of recurrences or improved prognosis of T1RC patients. However, the optimal surveillance intensity in terms of clinical outcomes remains to be elucidated.

The risk of recurrence after local resection seems higher for T1 cancers in the rectum compared to T1 cancers throughout the colon. Here, we found a risk of recurrence for rectal T1 cancers of 7.7% (after endoscopic resection or TEM/TAMIS), which is higher than the 3.3% for endoscopically treated T1 cancers at sites throughout the colorectum [9]. A similar difference was seen in the subgroup of low-risk (endoscopically treated T1 colorectal cancer: 0.7%; endoscopically or TEM/TAMIS-treated low-risk T1 rectal cancer: 3.1–5.9%) and high-risk cancers (endoscopically treated T1 colorectal cancer: 7.0%; endoscopically or TEM/TAMIS-treated low-risk T1 rectal cancer: 12.5–29.7%). These results suggest rectal T1 cancers are associated with worse outcomes compared to colonic T1 cancers, independent of histological risk status. Plausible contributing factors include differences in anatomic structures and tumor biology [119].

The most important limitation of this meta-analysis relates to the quality of the included studies. The selection of studies for this meta-analysis was performed as thoroughly as possible, to prevent the exclusion of important studies. However, several studies did not specifically study T1RC patients treated by local resection alone. Therefore, data on patient, treatment, tumor size, tumor height, histological, follow-up and individual recurrence characteristics could not always be fully extracted. This resulted in a smaller number of studies in various subgroup analyses and for some studies in not receiving the maximum assessment scores on risk of bias. Secondly, there was some statistical heterogeneity, which could be expected a priori considering the heterogeneity in the resection techniques and follow-up. Therefore, we performed extensive meta-regression and subgroup analyses, which yielded lower heterogeneity estimates. Lastly, the definition of the rectum was left to the discretion of the authors of included studies, to avoid exclusion of many relevant articles that did not clearly state a definition. However, due to the technical limitations of transanal local excisions proximal to the rectum, it is not likely that cancers outside the rectum were included in this meta-analysis.

## Clinical implications

**Fig. 6:**
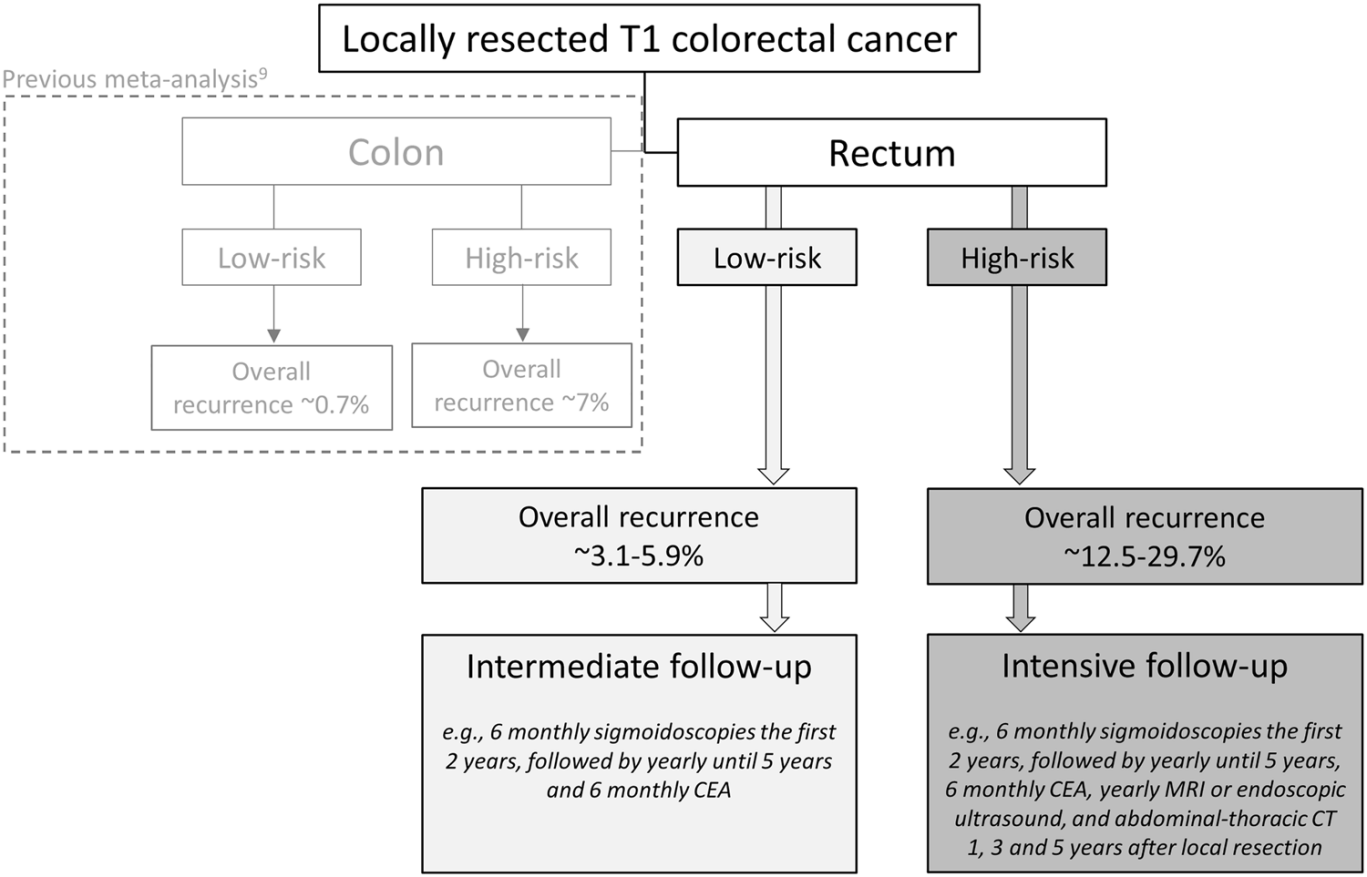
Overview of the main study findings and surveillance recommendations. *CEA* carcinoembryonic antigen, *MRI *magnetic resonance imaging, *CT* computed tomography

Based on our study findings, we propose the following surveillance recommendations and key points for future research. Firstly, T1RC patients should be offered a different follow-up than T1 colon cancer patients and the surveillance should be stratified for histological risk status. There is no need to stratify surveillance for local resection technique when the T1RC is removed endoscopically or by TEM/TAMIS. All T1RCs that are removed locally should be offered surveillance (provided that possible findings will have clinical consequences) because even for low-risk T1RCs the recurrence risk is 3.1–5.9%. Patients with locally resected low-risk T1RC should be offered surveillance rather than completion TME because we think that in these patients the potential drawbacks from oncological surgery are greater than the possible benefits. We propose a 5-year moderately intensive follow-up scheme that should focus on the local-endoluminal site where most recurrences seem to develop (e.g., 6 monthly (recto)sigmoidoscopies the first 2 years, and then yearly until 5 years; and 6 monthly CEA). Patients with high-risk T1RC should be offered completion TME surgery because of the relatively high-risk of recurrence, as is recommended in current guidelines [2, 120]. If oncological surgery is not feasible we propose a 5-year intensive follow-up scheme focusing on the detection of endoluminal, locoregional lymph node and distant recurrences (e.g., 6 monthly (recto)sigmoidoscopies the first 2 years, and then yearly until 5 years; 6 monthly CEA; yearly MRI or endoscopic ultrasound, and abdominal-thoracic computed tomography at 1, 3 and 5 years). This follow-up scheme only seems beneficial for those patients in whom salvage surgery or treatment of metastases seems feasible in the future. An overview of our main study findings and surveillance recommendations is shown in Fig. 6. Further prospective studies are necessary to study the optimal method, the optimal timing, cost-effectiveness of surveillance and the impact of surveillance on the prognosis.

## Conclusion

Patients with T1 rectal cancer may have a significantly lower recurrence risk after TEM/TAMIS compared to other local surgical resections. After TEM/TAMIS and endoscopic resection the recurrence risk, cancer-related mortality and cancer-related mortality among patients with recurrence were comparable. Recurrence was mainly dependent on histological risk status. Based on our findings we propose a more uniform histology-based surveillance strategy for T1 rectal cancer patients treated by local resection alone.

## Supplementary Information

Below is the link to the electronic supplementary material.

Supplementary methods and results (DOCX 51 kb)

Supplementary figure 1. Funnel plot for the cumulative incidence of any rectal cancer recurrence. The points correspond to the incidences of individual studies, and the vertical line in the funnel plot indicates the summary estimate. To visualize studies with 0 events, a continuity correction of +0.5 was applied (TIF 147486 kb)

Supplementary figure 2. Forest plot with cumulative incidences of any RC recurrence after local surgical resection in patients with ≥ 2 years follow-up. To visualize incidence estimates of studies with 0 events, a continuity correction of +0.5 was applied. Values of the pooled estimates, *I*^2^ and *τ*^2^ are calculated using a model without continuity correction (TIF 147486 kb)

Supplementary figure 3. Forest plot with cumulative incidences of rectal cancer-related mortality after local surgical resection in patients with ≥ 2 years follow-up. To visualize incidence estimates of studies with 0 events, a continuity correction of +0.5 was applied.Values of the pooled estimates, *I*^2^ and *τ*^2^ are calculated using a model without continuity correction (TIF 147486 kb)

Supplementary figure 4. Number of JSCCR criteria used for histological risk stratification for studies on TEM/TAMIS and endoscopic resection. *TEM* transanal endoscopic microsurgery, *TAMIS* transanal minimally invasive surgery, *JSCCR* Japanese Society for Cancer of the Colon and Rectum (TIF 147486 kb)

Supplementary figure 5. Strictness of follow-up for studies on endoscopic resection and TEM/TAMIS. *TEM* transanal endoscopic microsurgery, *TAMIS* transanal minimally invasive surgery (TIF 147486 kb)

Supplementary figure 6. Time to any rectal cancer recurrence after endoscopic resection (TIF 147486 kb)

Supplementary figure 7. Time to any rectal cancer recurrence after local surgical resection (TIF 147486 kb)

Supplementary figure 8. Treatment of recurrence after local surgical resection. a. local endoluminal recurrence, b. locoregional recurrence, c. local/locoregional + distant recurrence, d. distant recurrence. *TME* total mesorectal excision, *CRT* chemoradiotherapy (TIF 2367 kb)

Supplementary figure 9. Time between rectal cancer recurrence and rectal cancer-related mortality (TIF 831 kb)

Supplementary table 1. In- and exclusion criteria for the endoscopic sub-section. *CRC *colorectal cancer, *RC* rectal cancer (DOCX 14 kb)

Supplementary table 2. Meta-regression with study characteristics and risk of bias. Potential predictors of statistical inter-study heterogeneity for the outcome "any rectal cancer recurrence". (DOCX 21 kb)

Supplementary table 3. Meta-regression with clinical characteristics. Potential predictors of statistical inter-study heterogeneity for the outcome "any rectal cancer recurrence". *LE* local excision, *TEM* transanal endoscopic microsurgery, *TAMIS* transanal minimally invasive surgery, *AV* anal verge, *DL* dentate line, *LVI *lymphovascular invasion (DOCX 20 kb)

Supplementary analyses (DOCX 11 kb)

Supplementary file: Prisma checklist (DOCX 31 kb)

## Data Availability

Data file, analytic methods and study materials will be made available in the supplementary data. This meta-analysis was not pre-registered.
