# Supplementary material for: Risk of recurrence after local resection of T1 rectal cancer: a meta-analysis with meta-regression
Source: Surg Endosc. 2022 Jun 30;36(12):9156–68. doi: 10.1007/s00464-022-09396-3 (PMC9652303; doi:10.1007/s00464-022-09396-3)
Supplement: Supplementary file 1 — Supplementary methods and results (DOCX 51 kb) [file 464_2022_9396_MOESM1_ESM.docx]

**Supplementary methods**

Search strategy

A systematic literature search was conducted in the electronic databases of PubMed, Embase, Web of Science and Cochrane Library from inception until May 19, 2021. The strategy included terms for “T1 rectal cancer”, “local surgical resection” and “recurrence”. Duplicates were removed and the eligibility of the studies was independently assessed by 3 authors (ND/HD/JK). A fourth assessor (JB) was decisive in case of disagreement after discussion.

The detailed search strategies per database are shown below.

*PubMed: n =1988 hits on May 19, 202*

("Colorectal Neoplasms"[Mesh] OR (("Intestine, Large"[Mesh] OR large intestin*[tw] OR "Colon"[tw] OR "colonic"[tw] OR "colorectal"[tw] OR "Rectum"[tw] OR "rectal"[tw] OR "cecum"[tw] OR "coecum"[tw] OR "cecal"[tw] OR "coecal"[tw] OR "large bowel"[tw] OR lower gastro*[tw]) AND ("Neoplasms"[Mesh:NoExp] OR Neoplas*[tw] OR "Carcinoma"[Mesh:NoExp] OR carcinoma*[tw] OR "Adenocarcinoma"[Mesh:NoExp] OR Adenocarcinoma*[tw] OR "cancer"[tw] OR "cancers"[tw] OR "Polyps"[Mesh:NoExp] OR "Intestinal Polyps"[Mesh] OR "polyp"[tw] OR "polyps"[tw] OR "tumor"[tw] OR "tumors"[tw] OR "tumour"[tw] OR "tumours"[tw] OR malignan*[tw] OR dysplas*[tw]))) AND ("T1"[tw] OR "T 1"[tw] OR "cT1"[tw] OR "c T1"[tw] OR "pT1"[tw] OR "p T1"[tw] OR "early"[ti] OR "stage I"[tw] OR "stage 1"[tw] OR "stage1"[tw] OR submucosa*[tw] OR "Dukes A"[tw] OR "Stage A"[tw]) AND ("Transanal Endoscopic Microsurgery"[Mesh] OR "transanal endoscopic microsurgery"[tw] OR "transanal endoscopic microsurg*"[tw] OR "trans anal endoscopic microsurgery"[tw] OR "trans anal endoscopic microsurg*"[tw] OR (("transanal"[tw] OR transanal*[tw] OR "trans-anal"[tw] OR trans-anal*[tw]) AND ("microsurgery"[tw] OR "microsurg*"[tw] OR "micro surgery"[tw] OR "micro surg*"[tw] OR "Microsurgery"[Mesh])) OR "Transanal Endoscopic Surgery"[mesh] OR "transanal minimally invasive surgery"[tw] OR "transanal minimal invasive surgery"[tw] OR "transanal minimally invasive"[tw] OR "transanal minimal invasive"[tw] OR (("transanal"[tw] OR transanal*[tw] OR "trans-anal"[tw] OR trans-anal*[tw]) AND ("Minimally Invasive Surgical Procedures"[mesh:noexp] OR "minimal invasive"[tw] OR "minimally invasive"[tw])) OR "TAMIS"[tiab] OR rectoscop*[tw] OR "Proctoscopy"[Mesh] OR proctoscop*[tw] OR polypect*[tw] OR (("locally"[tw] OR "local"[tw] OR transana*[tw] OR "full thickness"[tw] OR "full-thickness"[tw]) AND (resect*[tw] OR dissect*[tw] OR excis*[tw]))) AND ("Recurrence"[Mesh] OR "Neoplasm Recurrence, Local"[Mesh] OR Recurren*[tw] OR "Disease Progression"[Mesh:NoExp] OR progress*[tw] OR relaps*[tw] OR reoccur*[tw] OR reappear*[tw] OR return*[tw] OR "Neoplasm, Residual"[Mesh] OR residual*[tw] OR incomplete resect*[tw] OR "Reoperation"[Mesh] OR Reoperat*[tw] OR "Neoplasm Metastasis"[Mesh] OR metastas*[tw] OR "metastatic"[tw]) NOT ("Case Reports" [Publication Type] OR "case report"[ti] OR "case reports"[ti]) NOT ("Animals"[Mesh] NOT "Humans"[Mesh])

*Embase: n = 1726 hits on May 19, 2021*

(colon tumor/ or exp colon cancer/ or colon polyp/ or colorectal tumor/ OR rectum tumor/ OR exp rectum cancer/ OR rectum polyp/ OR ((exp large intestine/ OR large intestin*.ti,ab. OR "cecum".ti,ab. OR "coecum".ti,ab. OR "cecal".ti,ab. OR "coecal".ti,ab. OR "Colon".ti,ab. OR "colonic".ti,ab. OR "colorectal".ti,ab. OR "Rectum".ti,ab. OR "rectal".ti,ab. OR "large bowel".ti,ab. OR lower gastro*.ti,ab.) AND (neoplasm/ OR Neoplas*.ti,ab. OR carcinoma/ OR carcinoma*.ti,ab. OR adenocarcinoma/ OR Adenocarcinoma*.ti,ab. OR "cancer".ti,ab. OR "cancers".ti,ab. OR polyp/ OR exp intestine polyp/ OR "polyp".ti,ab. OR "polyps".ti,ab. OR "tumor".ti,ab. OR "tumors".ti,ab. OR "tumour".ti,ab. OR "tumours".ti,ab. OR malignan*.ti,ab. OR malignant neoplasm/ OR dysplasia/ or gastrointestinal dysplasia/ OR dysplas*.ti,ab.))) AND ("T1".ti,ab. OR "T 1".ti,ab. OR "cT1".ti,ab. OR "c T1".ti,ab. OR "pT1".ti,ab. OR "p T1".ti,ab. OR "early".ti. OR "stage I".ti,ab. OR "stage 1".ti,ab. OR "stage1".ti,ab. OR submucosa/ OR submucosa*.ti,ab. OR "Dukes A".ti,ab. OR "Stage A".ti,ab.) AND ("Transanal Endoscopic Microsurgery"/ OR "transanal endoscopic microsurgery".ti,ab OR "transanal endoscopic microsurg*".ti,ab OR "trans anal endoscopic microsurgery".ti,ab OR "trans anal endoscopic microsurg*".ti,ab OR (("transanal".ti,ab OR transanal*.ti,ab OR "trans-anal".ti,ab OR trans-anal*.ti,ab) AND ("microsurgery".ti,ab OR "microsurg*".ti,ab OR "micro surgery".ti,ab OR "micro surg*".ti,ab OR exp "Microsurgery"/)) OR "transanal minimally invasive surgery".ti,ab OR "transanal minimal invasive surgery".ti,ab OR "transanal minimally invasive".ti,ab OR "transanal minimal invasive".ti,ab OR (("transanal".ti,ab OR transanal*.ti,ab OR "trans-anal".ti,ab OR trans-anal*.ti,ab) AND ("Minimally Invasive Surgery"/ OR "minimal invasive".ti,ab OR "minimally invasive".ti,ab)) OR "TAMIS".ti,ab OR rectoscopy/ OR rectoscop*.ti,ab. OR proctoscop*.ti,ab. OR polypectomy/ OR polypect*.ti,ab. OR local excision/ OR ((local therapy/ OR "locally".ti,ab. OR "local".ti,ab. OR "transanal*".ti,ab OR "full-thickness".ti,ab) AND (resect*.ti,ab. OR dissection/ OR dissect*.ti,ab. OR excision/ OR excis*.ti,ab.))) AND (recurrent disease/ OR tumor recurrence/ OR Recurren*.ti,ab. OR progress*.ti,ab. OR relapse/ OR relaps*.ti,ab. OR reoccur*.ti,ab. OR reappear*.ti,ab. OR return*.ti,ab. OR minimal residual disease/ OR residual*.ti,ab. OR incomplete resect*.ti,ab. OR reoperation/ OR Reoperat*.ti,ab. OR exp metastasis/ OR metastas*.ti,ab. OR "metastatic".ti,ab.) NOT (case report/ OR "case report".ti. OR "case reports".ti.) NOT (animal/ NOT human/) NOT ("conference abstract" OR "conference review").pt.

*Web of Science: n = 1947 hits on May 19, 2021*

TS=((large intestin* OR "Colon" OR "colonic" OR "colorectal" OR "Rectum" OR "rectal" OR "cecum" OR "coecum" OR "cecal" OR "coecal" OR "large bowel" OR lower gastro*) AND (Neoplas* OR carcinoma* OR Adenocarcinoma* OR "cancer" OR "cancers" OR "polyp" OR "polyps" OR "tumor" OR "tumors" OR "tumour" OR "tumours" OR malignan* OR dysplas*)) AND (TS=("T1" OR "T 1" OR "cT1" OR "c T1" OR "pT1" OR "p T1" OR "stage I" OR "stage 1" OR "stage1" OR submucosa* OR "Dukes A" OR "Stage A") OR TI="early") AND TS=("Transanal Endoscopic Microsurgery" OR "transanal endoscopic microsurgery" OR "transanal endoscopic microsurg*" OR "trans anal endoscopic microsurgery" OR "trans anal endoscopic microsurg*" OR (("transanal" OR transanal* OR "trans-anal" OR trans-anal*) AND ("microsurgery" OR "microsurg*" OR "micro surgery" OR "micro surg*" OR "Microsurgery")) OR "transanal minimally invasive surgery" OR "transanal minimal invasive surgery" OR "transanal minimally invasive" OR "transanal minimal invasive" OR (("transanal" OR transanal* OR "trans-anal" OR trans-anal*) AND ("Minimally Invasive Surgery" OR "minimal invasive" OR "minimally invasive")) OR "TAMIS" OR rectoscop* OR proctoscop* OR polypect* OR (("locally" OR "local" OR "transanal*" OR "full-thickness") AND (resect* OR dissect* OR excis*))) AND TS=(Recurren* OR progress* OR relaps* OR reoccur* OR reappear* OR return* OR residual* OR incomplete resect* OR Reoperat* OR metastas* OR "metastatic") NOT TI=("mouse" OR "mice" OR "murine" OR "rat" OR "rats" OR "animal" OR "animals" OR "rodent" OR "rodents")

Refined by: [excluding] DOCUMENT TYPES: (MEETING ABSTRACT)

Timespan: All years. Indexes: SCI-EXPANDED, SSCI, A&HCI, ESCI.

*Cochrane Library: n =249 hits on May 19, 2021*

(((large intestin* OR "Colon" OR "colonic" OR "colorectal" OR "Rectum" OR "rectal" OR "cecum" OR "coecum" OR "cecal" OR "coecal" OR "large bowel" OR lower gastro*) AND (Neoplas* OR carcinoma* OR Adenocarcinoma* OR "cancer" OR "cancers" OR "polyp" OR "polyps" OR "tumor" OR "tumors" OR "tumour" OR "tumours" OR malignan* OR dysplas*)) AND ("T1" OR "T 1" OR "cT1" OR "c T1" OR "pT1" OR "p T1" OR "stage I" OR "stage 1" OR "stage1" OR submucosa* OR "Dukes A" OR "Stage A" OR "early") AND ("Transanal Endoscopic Microsurgery" OR "transanal endoscopic microsurgery" OR "transanal endoscopic microsurg*" OR "trans anal endoscopic microsurgery" OR "trans anal endoscopic microsurg*" OR (("transanal" OR transanal* OR "trans anal" OR trans anal*) AND ("microsurgery" OR "microsurg*" OR "micro surgery" OR "micro surg*" OR "Microsurgery")) OR "transanal minimally invasive surgery" OR "transanal minimal invasive surgery" OR "transanal minimally invasive" OR "transanal minimal invasive" OR (("transanal" OR transanal* OR "trans anal" OR trans anal*) AND ("Minimally Invasive Surgery" OR "minimal invasive" OR "minimally invasive")) OR "TAMIS" OR rectoscop* OR proctoscop* OR polypect* OR (("locally" OR "local" OR "transanal" OR "transanal*" OR "full thickness") AND (resect* OR dissect* OR excis*))) AND (Recurren* OR progress* OR relaps* OR reoccur* OR reappear* OR return* OR residual* OR incomplete resect* OR Reoperat* OR metastas* OR "metastatic") NOT ("mouse" OR "mice" OR "murine" OR "rat" OR "rats" OR "animal" OR "animals" OR "rodent" OR "rodents")):ti,ab,kw NOT (meeting abstract OR conference abstract):

The search strategy for studies regarding the endoscopic resections for T1CRC was previously published(1). For this meta-analysis, the search was updated until May 19, 2021, the subset of patients with rectal lesions was selected and additional data regarding this subgroup was requested from corresponding authors.

Data extraction

The following data were extracted: study characteristics (year of publication, single- or multi-center, study design, inclusion period, geographical location) patient characteristics (number of patients undergoing local surgical resection for T1RC, sex, age, comorbidity), treatment characteristics (treatment modality used for local resection, resection plane, anatomical approach of the resection), tumor characteristics (size, distance to the anal verge/dentate line), histological characteristics (low- or high-risk T1RC, high-risk definition, resection margin status, number of patients with lymphovascular invasion (LVI), deep submucosal invasion, high-grade tumor budding, grade 3 differentiation), follow-up characteristics and outcomes (follow-up modalities used, frequency per follow-up modality, mean and minimum follow-up duration, number of locoregional or distant recurrences, RC-related mortality). For all individual recurrence cases, we also extracted available patient-level data on all aforementioned clinical characteristics, and recurrence management and outcomes.

For the subgroup of patients who were treated endoscopically, individual patient-level recurrence data, study characteristics, the total number of patients undergoing local endoscopic resection, follow-up characteristics, and outcomes were extracted. For practical reasons data on individual JSCCR features and follow-up duration specifically for T1RC were not collected.

When only median values and ranges were reported for continuous variables, the mean was estimated using the approximation method described by Luo and others(2).

Definitions and Classifications

Studies were classified as retrospective when this was explicitly or implicitly stated (e.g., “patient records were reviewed” or concluding that “prospective studies will be needed”). Studies that reported a retrospective analysis of prospectively collected data were classified as prospective. Studies were categorized as single-center when all authors were from 1 hospital and if it was not clearly stated in the manuscript how many centers the patients were included from. All four papers(3-6) in which discrepancies were found in between the text and tables/figures, were discussed in detail by two assessors (ND, HD) until consensus was reached.

The TEM and TAMIS techniques are well described in literature, first by Buess and Atallah(7, 8). The Gasless Transanal Endoscopic Surgery (GTES; (9)) and Video Endoscopic Transanal Rectal Tumour Excision (VTEM; (10)) were analyzed with the TEM group because of their similarities to TEM. Minimally Invasive Transanal Surgery (MITAS; (11)) was analyzed with TAMIS for the same reason. All other local surgical resection techniques using direct visualization (e.g., Parks technique, Stuart technique, the use of Ferguson’s anoscope, transanal endoscopic operation, or undefined local surgical resections) were grouped as “other local excisions”. Some papers reported more than one treatment modality. Patients for whom it was implicitly stated that they had undergone prior (endoscopic) resection (e.g., “no tumor rest was found in the specimen”) were excluded from this meta-analysis. However, for one study (12) it could not be ruled out that 1 of the 20 included patients was a case of T1 recurrence after endoscopic polypectomy. We chose to include this study to prevent loss of relevant data.

For each study, the high-risk definitions were classified based the number of JSCCR histological high-risk criteria that were used (i.e., grade 3 differentiation, deep submucosal invasion, high-grade tumor budding, LVI, and positive resection margins(13)). Grade 3 differentiation was defined as poorly differentiated adenocarcinoma, mucinous, or signet ring cell carcinoma(14). Deep submucosal invasion was defined as Kikuchi level ≥Sm2 or an invasion depth ≥1000 μm(15). High-grade tumor budding was defined as ≥Bd2(16). LVI was categorized into “present” or “absent” and was defined as CRC cells within an endothelial or internal elastic lamina-confined lumen(14). Resection margins were classified as R0 (no dysplastic cells at the resection margins), Rx (margins could not be assessed with certainty), or R1 (dysplastic cells close to or at the resection margins). Rx and R1 were then categorized as “not-R0” due to the fact that the exact number of Rx and R1 resections were often difficult to determine for the subgroup of interest and because of the varying definitions of R1 across the different studies.

Follow-up intensity was compared by grouping studies together based on the number of follow-up modalities used and the intervals per modality. Schemes with a mean of ≤2-3 modalities per year or only one modality (e.g., “3 monthly sigmoidoscopies for at least 5 years”(17)) were classified as “not strict”, schemes with a mean of 2-4 modalities per year and at least 2 different modalities used (e.g., “3 monthly colonoscopies and CEA for 2 years, followed by yearly for 5 years”(18)) as “strict” and schemes with ≥4 modalities per year and the use of at least 3 different modalities (e.g., “3 monthly proctoscopies, chest X-ray, abdominal ultrasound scanning, CEA and rectal intraluminal ultrasound for 2 years, followed by 6 monthly for 5 years”(3)) as “very strict”. When the follow-up duration was not reported separately for the subgroup of locally treated T1 patients, overall follow-up durations of the population that came closest to the population of interest (both in numbers and in treatment modality) were extracted and used in sensitivity analyses.

Risk of Bias Assessment

A modified Newcastle-Ottawa Scale for cohort studies(19) was used to assess the risk of bias. The items “Comparability of cohorts” and “Selection of the non-exposed cohort” were excluded because no comparative outcome measures were meta-analyzed. “Ascertain of exposure” was excluded because only patients with histologically confirmed T1RCs were included. The item “Was follow-up long enough for outcomes to occur” was adjusted to “Information on follow-up duration of analyzed patients with T1RC reported” (0, no; 1, yes). Reported follow-up durations were included in meta-regression analyses as continuous variables. “Information on follow-up intensity reported” was also added as an item (0, no; 1, yes; the number of modalities is reported; 2, yes; both the number of modalities and the frequency of use are reported; included as categorical variables in meta-regression analyses) because recurrence incidence, location, and time to recurrence largely depend on the number of follow-up modalities used and the frequency thereof. The influence of every individual risk of bias item on the study outcomes was evaluated using meta-regression analyses. It has been shown that the use of combined scores of all individual risk of bias items to identify the level of quality can be problematic(20). Therefore, no overall scores were included in our analyses.

Supplementary results

All additional forest plots are displayed in **Supplementary analyses**.

Individual recurrence cases

The type of recurrence was reported for 288 recurrence cases (192 locoregional, 43 distant, 53 both). The time to recurrence was reported in 152 cases(**Supplementary figure 7**). In 26 cases it was reported which modality detected the recurrence: 12 via endoscopy/endoscopic-ultrasound, 7 via magnetic resonance imaging (MRI), 5 via clinical exam, and 1 via serum Carcinoembryonic Antigen (CEA). The management of recurrences was reported in 153 cases(**Supplementary figure 8)**. Of the 100 patients with only endoluminal recurrence, most underwent additional TME (n=74) or a local re-excision (n=16), with or without (neo)adjuvant therapy. The ten other patients received (chemo)radiation therapy without resection, palliative care or no treatment. The T-stage of the recurrences was reported in 42 cases (9 T2; 28 T3; 3 T4). The N- and M-stage of recurrences were rarely reported. In 33 recurrence cases with data on both the time to recurrence and time to RC-related death, the median time from recurrence to RC-related death was 13 months (range, 0-104;**Supplementary figure 9**)**.**

Meta-regression

None of the study characteristics, risk of bias assessment items, and follow-up intensity were significantly associated with the risk of recurrence (all p>0.1) **Supplementary table 2**. Follow-up duration (15 studies) and reporting on follow-up scheme (reported vs. not reported, 86 studies) were not significant (p=0.06). In 59 studies a mean duration of follow-up was reported for a group of patients that included all locally treated T1 cancers but also included other patients (e.g., also including some patients receiving adjuvant therapies, T2 cancers, etc.). When this mean duration of follow-up was used in a sensitivity analysis a positive significant association between follow-up duration and risk of recurrence was found (p=0.0001). Therefore, further analyses were stratified according to the duration of follow-up. Of all clinical characteristics, histological risk profile showed the most significant association with the cumulative incidence of RC recurrence (p=0.0004), followed by treatment modality (p=0.0037 for LE vs. TEM/TAMIS). (**Supplementary table 3**).

Subgroup analyses stratified on follow-up duration

*All studies*

The pooled incidences of locoregional recurrence only, any locoregional recurrence and any distant recurrence were 6.5% (95%-CI 5.1-8.3%, I^2^ = 52.5%), 7.7% (95%-CI 6.0-9.8%, I^2^ = 66.5%) and 2.6% (95% CI 1.8-3.8%, I^2^ = 57.7%), respectively. The pooled incidence of RC-related mortality was 2.0% (55/1468 events, 52 studies; 95%-CI 1.1-3.8; I^2^ = 75.2%). The RC-related mortality rate among patients with recurrence was 31.4% (55/175). Besides one patient with direct postoperative mortality after salvage TME (patient ID 12571 no. 2; **Supplementary analyses; dataset**), all of these patients died of disease progression.

*≥ 2 years follow-up*

The pooled incidences of locoregional recurrence only, any locoregional recurrence and any distant recurrence were 6.5% (95%-CI 4.9-8.6% I^2^ = 43.1%), 7.5% (95%-CI 5.7-10.0%, I^2^ = 56.2%) and 3.1% (95%-CI 2.1-4.5%, I^2^ = 40.8%), respectively.

*≥ 5 years follow-up*

The pooled cumulative incidence of any RC recurrence was 13.2% (95%-CI 10.5-16.6%; I^2^ = 24.4%). The pooled incidences of locoregional recurrence only, any locoregional recurrence and any distant recurrence were 8.3% (95%-CI 6.7-10.3%, I^2^ = 0.0%), 10.3% (95%-CI 7.9-13.3%, I^2^ = 8.4%) and 4.9% (95%-CI 3.3-7.3%, I^2^ = 35.6%), respectively. The pooled incidence of RC-related mortality was 3.4% (22/489 events, 9 studies; 95%-CI 1.5-7.1%; I^2^ = 59.4%). The RC-related mortality rate among patients with recurrence was 31.4% (22/70). All of these patients died of disease progression.

Subgroup analyses stratified on histologic characteristics

*All studies*

The cumulative incidence of any T1RC recurrence was 7.1 % for low-risk T1RC (86/991 events; 95%-CI 5-9.9%; I^2^ = 48.8%) and 24.5% for high-risk T1RC (35/143 events; 95%-CI 18.1-32.2%; I^2^ = 0.0%). For low-risk patients, the pooled incidences of locoregional recurrence only, any locoregional recurrence and any distant recurrence were 4.4% (95% CI 3.2-6.1%, I^2^ = 0.0%), 5.3% (95%-CI 3.7-7.6%, I^2^ = 16.7%) and 2.1% (95%-CI 1.1-4.2%, I^2^ = 31.4%), respectively. For high-risk patients, the pooled incidences of locoregional recurrence only, any locoregional recurrence and any distant recurrence were 12.5% (95%-CI 7.7-19.7%, I^2^ = 5.4%), 20.7% (95%-CI 13.2-30.8%, I^2^ = 24.5%) and 11.9% (95%-CI 7.5-18.3%, I^2^= 0.0%), respectively. The pooled incidence of RC-related mortality was 1.2% for low-risk (16/596 events; 95%-CI 0.4-4.0%; I^2^ = 52.6%) and 0.8% for high-risk (4/89 events; 95%-CI 0.0-60%; I^2^ = 5.4%). The RC-related mortality rate among patients with recurrence was 33.3% (16/48) for low-risk and 17.4% (4/23) for high-risk. It was not possible to perform subgroup analyses based on low- and high-risk in combination with the number of used JSCCR criteria because of insufficient data.

*≥ 2 years follow-up*

The pooled incidences of locoregional recurrence only, any locoregional recurrence and any distant recurrence were 3.7% (95%-CI 2.5-5.5%, I^2^ = 0.0%), 4.8% (95%-CI 3.0-7.5%, I^2^ = 21.9%) and 2.8% (95%-CI 1.5-5.2%, I^2^ = 22.3%), respectively. For high-risk patients, the pooled incidences of locoregional recurrence only, any locoregional recurrence and any distant recurrence 16.9% (95% CI 9.9-27.4%, I^2^ = 0.0%), 23.7% (95%-CI 13.2-38.8%, I^2^ = 31.1%) and 11.3% (95%-CI 5.7-20.9%, I^2^= 0.0%), respectively. The pooled incidence of RC-related mortality was 0.9% for low-risk (3/340 events; 95%-CI 0.3-2.7%; I^2^ = 0.0%) and 23.1% (3/13) among low-risk patients with recurrence. Due to insufficient amount of studies the RC-related mortality could not be determined for high-risk. It was not possible to perform subgroup analyses based on low- and high-risk in combination with the number of used JSCCR criteria because of insufficient data.

*≥ 5 years follow-up*

The cumulative incidence of any T1RC recurrence was 9.1% for low-risk T1RC (24/265 events; 95%-CI 6.1-13.2%; I^2^ = 0.0%) and 24.6% for high-risk T1RC (15/61 events; 95%-CI 15.4-36.9%; I^2^ = 0.0%). For low-risk patients, the pooled incidences of locoregional recurrence only, any locoregional recurrence and any distant recurrence were 4.2% (95%-CI 2.3-7.3%, I^2^ = 0.0%), 6.1% (95% CI 3.2-11.5%, I^2^ = 7.9%) and 4.9% (95%-CI 2.9-8.3%, I^2^ = 0.0%), respectively. For high-risk patients, the pooled incidences of locoregional recurrence only, any locoregional recurrence and any distant recurrence were 13.1% (95%-CI 6.7-24.1%, I^2^ = 0.0%), 20.2% (95%-CI 9.8-37.1%, I^2^ = 31.3%) and 11.5% (95%-CI 5.6-22.2%, I^2^ = 0.0%), respectively. The pooled incidence of RC-related mortality was 10.0% for low-risk (1/105 events; 95%-CI 0.1-6.4%; I^2^ = 0.0%) and 16.7% (1/6) among low-risk patients with recurrence. Due to insufficient amount of studies the RC-related mortality could not be determined for high-risk. It was not possible to perform subgroup analyses based on low- and high-risk in combination with the number of used JSCCR criteria because of insufficient data.

Subgroup analyses stratified on treatment modality

*All studies*

The cumulative incidence of any T1RC recurrence was 6.9 % after TEM or TAMIS (135/1421 events; 95%-CI 4.9-9.6%; I^2^ = 66.4%) and 12.5% after other local excisions (124/810 events; 95%-CI 8.9-17.4%; I^2^ = 65.2%). For TEM/TAMIS, the pooled incidences of locoregional recurrence only, any locoregional recurrence and any distant recurrence were 5.3% (95%-CI 3.7-7.3%, I^2^ = 42.4%), 5.9% (95%-CI 4.0-8.8%, I^2^ = 70.4%) and 1.6% (95%-CI 0.8-3.3%, I^2^ = 74.2%), respectively. For other local excisions, the pooled incidences of locoregional recurrence only, any locoregional recurrence and any distant recurrence were 7.8% (95%-CI 5.0-11.9%, I^2^ = 58.2%), 9.6% (95%-CI 6.3-14.3%, I^2^ = 65.5%) and 4.0% (95%-CI 2.3-6.9%, I^2^ = 46.2%), respectively. The pooled incidence of RC-related mortality was 1.7% for TEM/TAMIS (24/788 events; 95%-CI 0.7-4.3%; I^2^ = 74.6%) and 4.1% for other local excisions (27/401 events; 95%-CI 1.7-9.5%; I^2^ = 66.4%). The RC-related mortality rate among patients with recurrence was 36.4% (24/66) for TEM/TAMIS and 40.3% (27/67) for other local excisions.

*≥ 2 years follow-up*

For TEM/TAMIS, the pooled incidences of locoregional recurrence only, any locoregional recurrence and any distant recurrence were 5.6% (95%-CI 3.7-8.4%, I^2^ = 23.5%), 6.4% (95%-CI 4.1-9.7%, I^2^ = 55.9%) and 2.3% (95%-CI 1.2-4.3%, I^2^ = 40.1%), respectively. For other local excisions, the pooled incidences of locoregional recurrence only, any locoregional recurrence and any distant recurrence were 7.0% (95%-CI 4-11.9%, I^2^ = 51.5%), 7.9% (95%-CI 4.6-13.4%, I^2^ = 61.0%) and 4.1% (95%-CI 2.1-8.0%, I^2^ = 48.6%), respectively. The pooled incidence of RC-related mortality was 2.8% for TEM/TAMIS (16/454 events; 95%-CI 1.2-6.2%; I^2^ = 48.9%) and 0.7% for other local excisions (11/209 events; 95%-CI 0.0-14.8%; I^2^ = 85.5%). The RC-related mortality rate among patients with recurrence was 35.6% (16/45) for TEM/TAMIS and 44% (11/24) for other local excisions.

*≥ 5 years follow-up*

The cumulative incidence of any T1RC recurrence was 12.2% after TEM or TAMIS (60/478 events; 95%-CI 8.8-16.8%; I^2^ = 10.4%) and 14.6% after other local excisions (61/400 events; 95%-CI 10.4-20.1%; I^2^ = 28.3%). For TEM/TAMIS, the pooled incidences of locoregional recurrence only, any locoregional recurrence and any distant recurrence were 7.5% (95%-CI 5.4-10.3%, I^2^ = 0.0%), 9.6% (95%-CI 6.0-15.0%, I^2^ = 38.2%) and 4.6% (95%-CI 2.7-8.0%, I^2^= 14.7%), respectively. For other local excisions, the pooled incidences of locoregional recurrence only, any locoregional recurrence and any distant recurrence were 8.9% (95%-CI 6.3-12.5%, I^2^ = 0.0%), 9.8% (95%-CI 7.0-13.6%, I^2^ = 0.0%) and 6.0% (95%-CI 3.1-11.3%, I^2^ = 40.4%), respectively. The pooled incidence of RC-related mortality was 3.3% for TEM or TAMIS (8/242 events; 95%-CI 1.7-6.5%; I^2^ = 0%) and 7.7% for other local excisions (11/109 events; 95%-CI 1.8-27.7%; I^2^ = 31.6%). The RC-related mortality rate among patients with recurrence was 27.6% (8/29) for TEM/TAMIS and 47.8% (11/23) for other local excisions.

Secondary outcomes after local endoscopic resection

After local endoscopic resections the pooled incidences of locoregional recurrence only, any locoregional recurrence and any distant recurrence were 4.7% (95%-CI 3.3-6.6%, I^2^ = 0.0%), 4.8% (95%-CI 2.5-9.0%, I^2^ = 67.1%) and 3.3% (95%-CI 2.1-5.1%, I^2^ = 6.5%), respectively. Due to insufficient data it was not possible to perform more in-depth subgroup analyses.

*All studies*

When combining TEM/TAMIS and local endoscopic resections, the cumulative incidence of any T1RC recurrence was 7.1% (186/2062 events; 95%-CI 5.4-9.3%; I^2^ = 62.4%). The pooled incidences of locoregional recurrence only, any locoregional recurrence and any distant recurrence were 5.1% (95%-CI 3.8-6.6%, I^2^ = 39.7%), 5.6% (95%-CI 3.9-7.8%, I^2^ = 70.9%) and 2.2% (95%-CI 1.3-3.5%, I^2^ = 63.7%), respectively. The pooled incidence of RC-related mortality was 2.0% (33/1167 events; 95%-CI 1.0-3.8%; I^2^ = 64.1%). The RC-related mortality rate among patients with recurrence was 48.3% (29/60).

*≥ 2 years follow-up*

When combining TEM/TAMIS and local endoscopic resections, the pooled incidences of locoregional recurrence only, any locoregional recurrence and any distant recurrence were 5.2% (95%-CI 3.9-6.9%, I^2^ = 24.1%), 5.7% (95%-CI 4.0-8.1%, I^2^ = 62.2%) and 2.8% (95%-CI 1.9-4.1%, I^2^ = 31.8%), respectively. The pooled incidence of RC-related mortality was 2.6% (25/833 events; 95%-CI 1.4-4.6%; I^2^ = 42.4%). The RC-related mortality rate among patients with recurrence was 33.3% (25/75).

*≥ 5 years follow-up*

When combining TEM/TAMIS and local endoscopic resections, the pooled incidence of any T1RC recurrence was 9.2% (111/1119 events; 95%-CI 7.0-12.0%; I^2^ = 43.1%) .The pooled incidences of locoregional recurrence only, any locoregional recurrence and any distant recurrence were 5.6% (95% CI 4.1-7.6%, I^2^ = 20.7%), 6.3% (95%-CI 4.2-9.5%, I^2^ = 65.4%) and 3.7% (95%-CI 2.6-5.4%, I^2^ = 24.1%), respectively. The pooled incidence of RC-related mortality was 2.7% (17/621 events; 95%-CI 1.7-4.4%; I^2^ = 2.9%). The RC-related mortality rate among patients with recurrence was 28.8% (17/59).

**References**

1. Dang H, Dekkers N, le Cessie S, et al. Risk and Time Pattern of Recurrences After Local Endoscopic Resection of T1 Colorectal Cancer: A Meta-analysis. Clinical Gastroenterology and Hepatology. 2020.

2. Luo D, Wan X, Liu J, et al. Optimally estimating the sample mean from the sample size, median, mid-range, and/or mid-quartile range. Stat Methods Med Res. 2018;27(6):1785-805.

3. Guerrieri M, Gesuita R, Ghiselli R, et al. Treatment of rectal cancer by transanal endoscopic microsurgery: experience with 425 patients. World J Gastroenterol. 2014;20(28):9556-63.

4. Taylor RH, Hay JH, Larsson SN. Transanal local excision of selected low rectal cancers. Am J Surg. 1998;175(5):360-3.

5. Ptok H, Marusch F, Meyer F, et al. Oncological outcome of local vs radical resection of low-risk pT1 rectal cancer. Arch Surg. 2007;142(7):649-55; discussion 56.

6. Balyasnikova S, Read J, Tait D, et al. The results of local excision with or without postoperative adjuvant chemoradiotherapy for early rectal cancer among patients choosing to avoid radical surgery. Colorectal Dis. 2017;19(2):139-47.

7. Buess G, Hutterer F, Theiss J, et al. [A system for a transanal endoscopic rectum operation]. Chirurg. 1984;55(10):677-80.

8. Atallah S, Albert M, Larach S. Transanal minimally invasive surgery: a giant leap forward. Surg Endosc. 2010;24(9):2200-5.

9. Vorobiev GI, Tsarkov PV, Sorokin EV. Gasless transanal endoscopic surgery for rectal adenomas and early carcinomas. Tech Coloproctol. 2006;10(4):277-81.

10. Nakagoe T, Ishikawa H, Sawai T, et al. Surgical technique and outcome of gasless video endoscopic transanal rectal tumour excision. Br J Surg. 2002;89(6):769-74.

11. Maeda K, Maruta M, Sato H, et al. Outcomes of novel transanal operation for selected tumors in the rectum. J Am Coll Surg. 2004;199(3):353-60.

12. Ganai S, Kanumuri P, Rao RS, et al. Local recurrence after transanal endoscopic microsurgery for rectal polyps and early cancers. Ann Surg Oncol. 2006;13(4):547-56.

13. Hashiguchi Y, Muro K, Saito Y, et al. Japanese Society for Cancer of the Colon and Rectum (JSCCR) guidelines 2019 for the treatment of colorectal cancer. International Journal of Clinical Oncology. 2020;25(1):1-42.

14. Compton CC, Fielding LP, Burgart LJ, et al. Prognostic factors in colorectal cancer. College of American Pathologists Consensus Statement 1999. Arch Pathol Lab Med. 2000;124(7):979-94.

15. Kitajima K, Fujimori T, Fujii S, et al. Correlations between lymph node metastasis and depth of submucosal invasion in submucosal invasive colorectal carcinoma: a Japanese collaborative study. J Gastroenterol. 2004;39(6):534-43.

16. Lugli A, Kirsch R, Ajioka Y, et al. Recommendations for reporting tumor budding in colorectal cancer based on the International Tumor Budding Consensus Conference (ITBCC) 2016. Mod Pathol. 2017;30(9):1299-311.

17. Budhoo, Hancock. Transanal excision of early rectal carcinoma-review of a personal series. Colorectal Dis. 2000;2(2):73-6.

18. Pakkastie T, Järvinen HJ. Local excision as an option in the treatment of low rectal carcinoma. Ann Chir Gynaecol. 1997;86(4):291-6.

19. Wells GA SB, O'Connell D, et al. The Newcastle-Ottawa Scale (NOS) for assessing the quality of nonrandomised studies in meta-analyses. Date accessed: April 6, 2020. [Available from: <http://www.ohri.ca/programs/clinical_epidemiology/oxford.htm>

20. Jüni P, Witschi A, Bloch R, et al. The Hazards of Scoring the Quality of Clinical Trials for Meta-analysis. JAMA. 1999;282(11):1054-60.
