# Supplementary figures and images for: Risk of recurrence after local resection of T1 rectal cancer: a meta-analysis with meta-regression
Source: Surg Endosc. 2022 Jun 30;36(12):9156–68. doi: 10.1007/s00464-022-09396-3 (PMC9652303; doi:10.1007/s00464-022-09396-3)

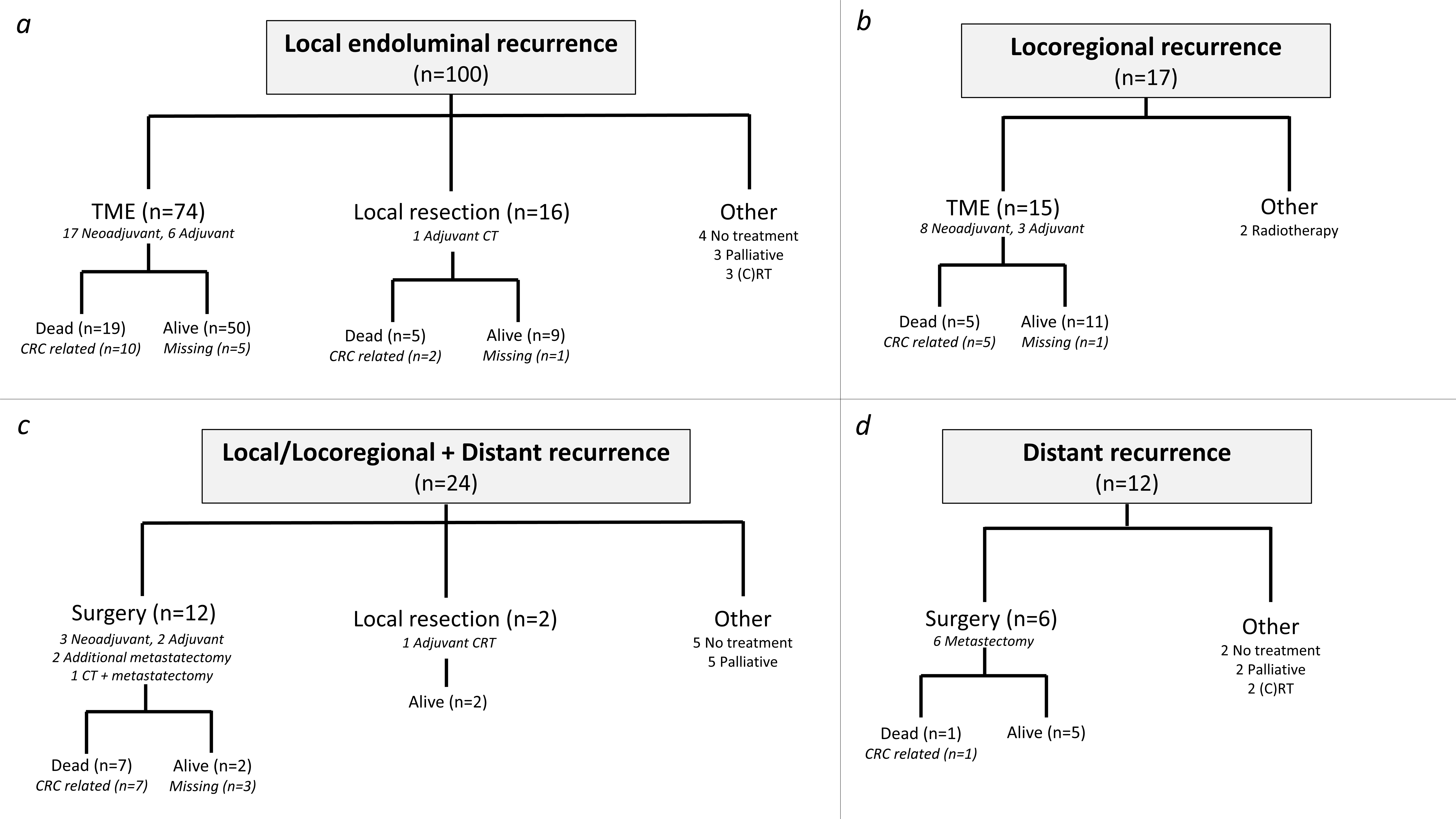

Supplement: Supplementary file 9 — Supplementary figure 8. Treatment of recurrence after local surgical resection. a. local endoluminal recurrence, b. locoregional recurrence, c. local/locoregional + distant recurrence, d. distant recurrence. TME total mesorectal excision, CRT chemoradiotherapy (TIF 2367 kb) [file 464_2022_9396_MOESM9_ESM.tif]

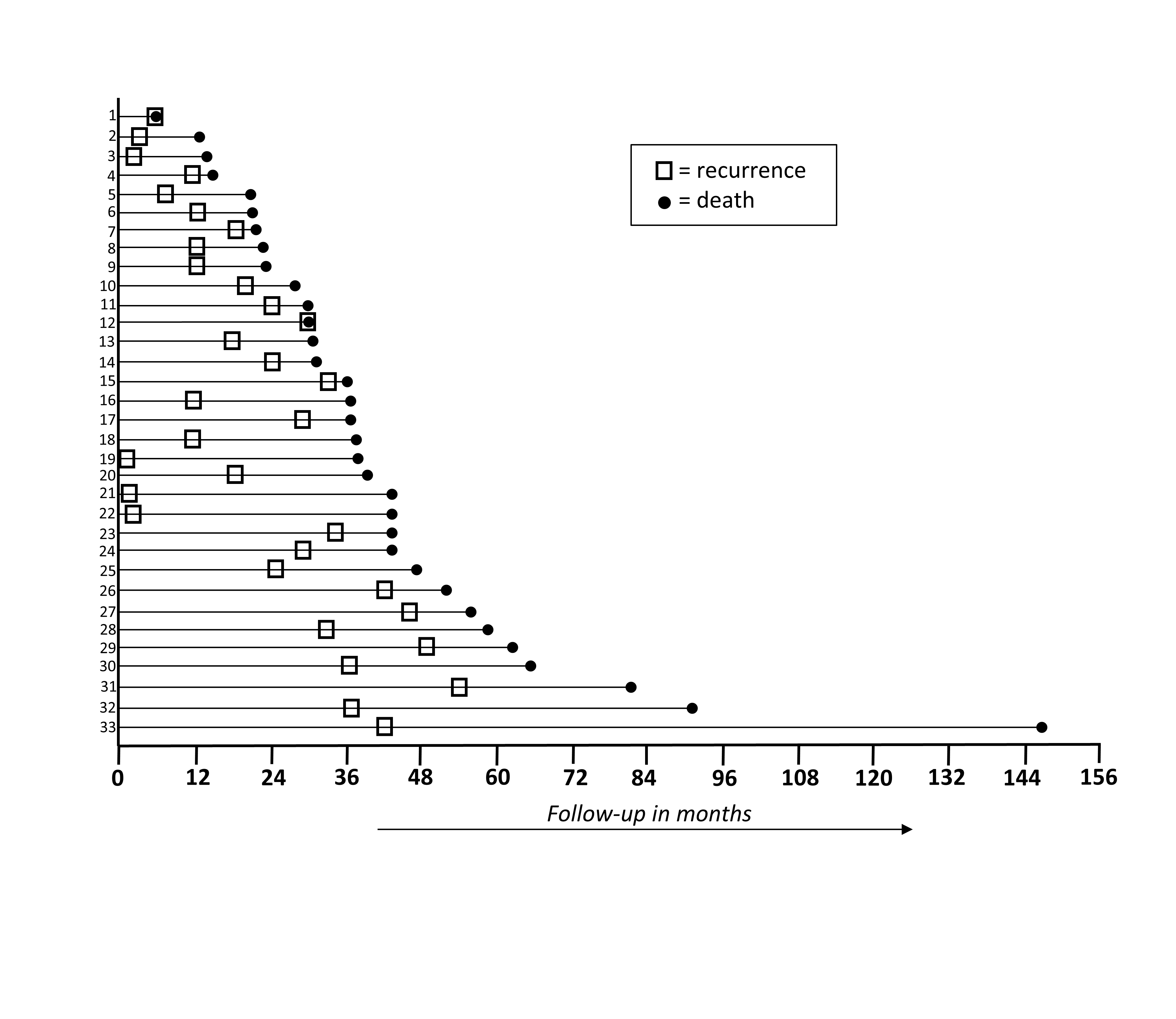

Supplement: Supplementary file 10 — Supplementary figure 9. Time between rectal cancer recurrence and rectal cancer-related mortality (TIF 831 kb) [file 464_2022_9396_MOESM10_ESM.tif]
