# Supplementary material for: Risk of recurrence after local resection of T1 rectal cancer: a meta-analysis with meta-regression
Source: Surg Endosc. 2022 Jun 30;36(12):9156–68. doi: 10.1007/s00464-022-09396-3 (PMC9652303; doi:10.1007/s00464-022-09396-3)
Supplement: Supplementary file 11 — Supplementary table 1. In- and exclusion criteria for the endoscopic sub-section. CRC colorectal cancer, RC rectal cancer (DOCX 14 kb) [file 464_2022_9396_MOESM11_ESM.docx]

| **Inclusion criteria previous meta-analysis** | **Exclusion criteria previous meta-analysis** |
| --- | --- |
| 1. Histologically confirmed patients with T1CRC treated with endoscopic resection alone | 1. Surgically treated patients with T1CRC |
| 1. Proportion of CRC recurrences reported for endoscopically treated patients with T1CRC | 2. Down-staged T1CRC after neoadjuvant therapy |
| 1. Originally peer-reviewed articles | 1. Patients receiving adjuvant therapy |
|  | 1. Hereditary predisposition for CRC |
|  | 1. Inflammatory bowel disease |
|  | 1. Case report studies with <5 patients |
|  | 1. Studies without original patient data |
|  | 1. Conference articles |
|  | 1. Animal studies |
| Endoscopic sub-section of current meta-analysis:  Identical criteria when “T1CRC” is replaced with “T1RC” | |

**Supplementary Table 1**. In- and exclusion criteria for the endoscopic sub-section
