# Supplementary material for: Risk of recurrence after local resection of T1 rectal cancer: a meta-analysis with meta-regression
Source: Surg Endosc. 2022 Jun 30;36(12):9156–68. doi: 10.1007/s00464-022-09396-3 (PMC9652303; doi:10.1007/s00464-022-09396-3)
Supplement: Supplementary file 12 — Supplementary table 2. Meta-regression with study characteristics and risk of bias. Potential predictors of statistical inter-study heterogeneity for the outcome "any rectal cancer recurrence". (DOCX 21 kb) [file 464_2022_9396_MOESM12_ESM.docx]

**Supplementary table 2**: Meta-regression with study characteristics and risk of bias assessment.

| **Variable** | **Number of  T1 CRC patients** | **Number of studies** | **I^2^ overall** | **Overall heterogeneity(τ^2^_total_)** | **Coefficient variable** | **Residual heterogeneity (τ^2^_unexplained_)** | **Heterogeneity explained by variable (R^2^)** | **p-value** |
| --- | --- | --- | --- | --- | --- | --- | --- | --- |
| *Study characteristics* | | | | | | | | |
| Publication year | 2585 | 86 | 68.3% | 0.5370 | 0.0239 | 0.5133 | 4.413408% | 0.1360 |
| Single vs. multicenter | 2471 | 84 | 68.673% | 0.5479 | 0.1616 | 0.5520 | 0% | 0.5522 |
| Retrospective vs. prospective | 2481 | 81 | 69.1696% | 0.5370 | -0.3954 | 0.4853 | 9.62756% | 0.0883 |
| Geographical location (i.e. continent) | 2585 | 86 | 68.3% | 0.5370 | 0.1221 | 0.5345 | 0.465549% | 0.2745 |
| *Risk of bias assessment items* | | | | | | | | |
| Signs of selective inclusion | 2585 | 86 | 68.3% | 0.5370 | *NA* | *NA* | *NA* | *NA* |
| Outcome not present at baseline  (i.e. patients with signs of distant disease excluded) | 2585 | 86 | 68.3% | 0.5370 | 0.2919 | 0.5229 | 2.6257% | 0.2227 |
| Outcome assessment | 2585 | 86 | 68.3% | 0.5370 | *NA* | *NA* | *NA* | *NA* |
| Adequacy of cohort follow-up | 2585 | 86 | 68.3% | 0.5370 | 0.3409 | 0.5094 | 5.1397% | 0.1316 |
| Data on follow-up duration reported ? | 2585 | 86 | 68.3% | 0.5370 | -0.1192 | 0.5264 | 1.97393% | 0.6114 |
| Data on follow-up scheme reported ? (i.e. which modalities used, timing per modality) | 2585 | 86 | 68.3% | 0.5370 | -0.2449 | 0.4800 | 10.61452% | 0.0690 |
| *Follow-up characteristics* |  |  |  |  |  |  |  |  |
| Mean follow-up duration | 494 | 15 | 0.0% | 0 | 0.0210 | 0 | 0% | 0.0693 |
| **Sensitivity analysis with mean follow- up duration of whole study group**  **“Mean sensitivity”** | **1812** | **59** | **60.8225%** | **0.3937** | **0.0158** | **0.1573** | **60.0457%** | **0.0001** |
| Minimum follow-up duration | 1515 | 51 | 53.5836% | 0.3024 | 0.0091 | 0.2852 | 5.6878% | 0.2114 |
| Number of follow-up modalities used | 1448 | 49 | 61.3896% | 0.4733 | 0.2136 | 0.4532 | 4.2468% | 0.1716 |
| Follow-up intensity | 1327 | 42 | 66.856% | 0.5582 | 0.0574 | 0.5547 | 0.627% | 0.8234 |
