# Supplementary material for: Risk of recurrence after local resection of T1 rectal cancer: a meta-analysis with meta-regression
Source: Surg Endosc. 2022 Jun 30;36(12):9156–68. doi: 10.1007/s00464-022-09396-3 (PMC9652303; doi:10.1007/s00464-022-09396-3)
Supplement: Supplementary file 13 — Supplementary table 3. Meta-regression with clinical characteristics. Potential predictors of statistical inter-study heterogeneity for the outcome "any rectal cancer recurrence". LE local excision, TEM transanal endoscopic microsurgery, TAMIS transanal minimally invasive surgery, AV anal verge, DL dentate line, LVI lymphovascular invasion (DOCX 20 kb) [file 464_2022_9396_MOESM13_ESM.docx]

**Supplementary table 3.** Meta-regression analysis with clinical characteristics

| **Variable** | **Number of  T1 CRC patients** | **Number of studies** | **I^2^ overall** | **Overall heterogeneity(τ^2^_total_)** | **Coefficient variable** | **Residual heterogeneity (τ^2^_unexplained_)** | **Heterogeneity explained by variable (R^2^)** | **p-value** |
| --- | --- | --- | --- | --- | --- | --- | --- | --- |
| *Patient characteristics* | | | | | | | | |
| Mean age | 571 | 14 | 74.7144% | 0.4856 | -0.0032 | 0.4865 | 0% | 0.9585 |
| Percentage males | 619 | 15 | 57.0864% | 0.2114 | 1.9051 | 0.1691 | 20.00946% | 0.1543 |
| *Treatment characteristics* |  |  |  |  |  |  |  |  |
| **Treatment (LE/LE+TEM/TEM/TAMIS)** | **2585** | **86** | **68.3%** | **0.5370** | **-0.3003** | **0.4306** | **19.814%** | **0.0084** |
| **Treatment (LE/TEM+TAMIS)** | **2585** | **86** | **68.3%** | **0.5370** | **-0.6235** | **0.4362** | **18.771%** | **0.0037** |
| Treatment (TEM vs TAMIS only) | 1385 | 45 | 67.5943% | 0.5731 | 0.4233 | 0.5745 | 0% | 0.5470 |
| *Tumor characteristics* |  |  |  |  |  |  |  |  |
| *Maximal distance in cm from AV/DL | 591 | 18 | 59.7128% | 0.3099 | 0.0690 | 0.3540 | 0% | 0.3104 |
| Mean tumor size in mm | 424 | 10 | 61.572% | 0.2569 | -0.0003 | 0.2577 | 0% | 0.9927 |
| *Maximal tumor size in mm | 434 | 15 | 80.473% | 1.2959 | 0.0371 | 0.7042 | 45.6594% | 0.1026 |
| **High/Low-risk** | **1167** | **48** | **56.803%** | **0.4652** | **1.4307** | **0.2613** | **43.8306%** | **0.0004** |
| **Not R0** | **1251** | **47** | **48.816%** | **0.2839** | **5.1151** | **0.2302** | **18.9151%** | **0.00297** |
| LVI proportion | 773 | 29 | 47.041% | 0.2949 | -5.2947 | 0.3056 | 0% | 0.5198 |
| Poor differentiation | 1267 | 43 | 65.2917% | 0.5090 | 7.3940 | 0.4694 | 7.77996% | 0.0561 |
| **Deep invasion** | **575** | **17** | **68.221%** | **0.4210** | **1.7168** | **0.1839** | **56.3183%** | **0.0134** |
