# Supplementary material for: Risk of recurrence after local resection of T1 rectal cancer: a meta-analysis with meta-regression
Source: Surg Endosc. 2022 Jun 30;36(12):9156–68. doi: 10.1007/s00464-022-09396-3 (PMC9652303; doi:10.1007/s00464-022-09396-3)
Supplement: Supplementary file 14 — Supplementary analyses (DOCX 11 kb) [file 464_2022_9396_MOESM14_ESM.docx]

Link to **Supplementary analyses**

<https://drive.google.com/file/d/1SFFnOpmPik8TTODVNWxlV5flqTFpr_Ta/view?usp=sharing>
